# Strategies of chemolithoautotrophs adapting to high temperature and extremely acidic conditions in a shallow hydrothermal ecosystem

**DOI:** 10.1186/s40168-023-01712-w

**Published:** 2023-12-05

**Authors:** Wenchao Deng, Zihao Zhao, Yufang Li, Rongguang Cao, Mingming Chen, Kai Tang, Deli Wang, Wei Fan, Anyi Hu, Guangcheng Chen, Chen-Tung Arthur Chen, Yao Zhang

**Affiliations:** 1 State Key Laboratory of Marine Environmental Sciences, Xiamen University, Xiamen, 361101 China; 2 Key Laboratory of Marine Ecological Conservation and Restoration, Third Institute of Oceanography, Ministry of Natural Resources, Xiamen, 361005 China; 3 Department of Functional and Evolutionary Ecology, Bio-Oceanography and Marine Biology Unit, University of Vienna, Djerassiplatz 1, 1030 Vienna, Austria; 4 Fisheries College, Jimei University, Xiamen, 361021 China; 5 Ocean College, Zhejiang University, Zhoushan, 316000 China; 6 CAS Key Laboratory of Urban Pollutant Conversion, Institute of Urban Environment, Chinese Academy of Sciences, Xiamen, 361021 China; 7 Department of Oceanography, National Sun Yat-Sen University, Kaohsiung Taiwan, China

**Keywords:** Shallow-sea hydrothermal vent, Chemolithoautotroph, Metabolic function, Adaptation mechanism, High temperature, Low pH, Stable isotope probing

## Abstract

**Background:**

Active hydrothermal vents create extreme conditions characterized by high temperatures, low pH levels, and elevated concentrations of heavy metals and other trace elements. These conditions support unique ecosystems where chemolithoautotrophs serve as primary producers. The steep temperature and pH gradients from the vent mouth to its periphery provide a wide range of microhabitats for these specialized microorganisms. However, their metabolic functions, adaptations in response to these gradients, and coping mechanisms under extreme conditions remain areas of limited knowledge. In this study, we conducted temperature gradient incubations of hydrothermal fluids from moderate (pH = 5.6) and extremely (pH = 2.2) acidic vents. Combining the DNA-stable isotope probing technique and subsequent metagenomics, we identified active chemolithoautotrophs under different temperature and pH conditions and analyzed their specific metabolic mechanisms.

**Results:**

We found that the carbon fixation activities of *Nautiliales* in vent fluids were significantly increased from 45 to 65 °C under moderately acidic condition, while their heat tolerance was reduced under extremely acidic conditions. In contrast, *Campylobacterales* actively fixed carbon under both moderately and extremely acidic conditions under 30 − 45 °C. Compared to *Campylobacterales*, *Nautiliales* were found to lack the Sox sulfur oxidation system and instead use NAD(H)-linked glutamate dehydrogenase to boost the reverse tricarboxylic acid (rTCA) cycle. Additionally, they exhibit a high genetic potential for high activity of cytochrome bd ubiquinol oxidase in oxygen respiration and hydrogen oxidation at high temperatures. In terms of high-temperature adaption, the *rgy* gene plays a critical role in *Nautiliales* by maintaining DNA stability at high temperature. Genes encoding proteins involved in proton export, including the membrane arm subunits of proton-pumping NADH: ubiquinone oxidoreductase, K^+^ accumulation, selective transport of charged molecules, permease regulation, and formation of the permeability barrier of bacterial outer membranes, play essential roles in enabling *Campylobacterales* to adapt to extremely acidic conditions.

**Conclusions:**

Our study provides in-depth insights into how high temperature and low pH impact the metabolic processes of energy and main elements in chemolithoautotrophs living in hydrothermal ecosystems, as well as the mechanisms they use to adapt to the extreme hydrothermal conditions.

Video Abstract

**Supplementary Information:**

The online version contains supplementary material available at 10.1186/s40168-023-01712-w.

## Background

Hydrothermal vents form in volcanically active areas, where Earth’s tectonic plates are spreading apart and where magma rises to the surface or close beneath the seafloor. Ocean water percolates into the crust through cracks and porous rocks and is heated by underlying magma, then the heated water reacts with hot rocks, enriching it with various chemicals and volatile gases. This hot buoyant hydrothermal fluid rises and emerges from vents in the sea floor, and rapidly mixes with cold seawater to form the final hydrothermal fluids [1]. Hydrothermal fluids are usually enriched in reduced components, such as hydrogen sulfide (H_2_S), methane (CH_4_), and dihydrogen (H_2_) [2–4]. Chemolithoautotrophs convert CO_2_ to organic carbon using the energy produced by oxidizing these reduced compounds [5]. In turn, this organic carbon support lush communities of animals that live around the active hydrothermal vents through symbiotic relationships with bacteria via grazing or suspension feeding, followed by trophic transfer [1]. As the primary producer of hydrothermal vent ecosystems, chemolithoautotrophs have received much attention since the first discovery of deep-sea hydrothermal ecosystems nearly 40 years ago [6].

Hydrothermal vents are typically sulfur-rich [7]. Previous investigations have found that chemolithoautotrophs are mainly classified in *Campylobacteria* (previously termed *Epsilonproteobacteria* [8]), *Gammaproteobacteria*, *Aquificae*, and some archaeal taxa [3, 9, 10]. They couple the carbon, sulfur, and nitrogen cycles together when fixing carbon in these unique ecosystems [11–13]. From the center to the outside of a vent, the microbial communities usually change significantly through 16S rRNA gene analysis [14, 15]. Typically, the abundance of *Epsilonproteobacteria* decreases with the increase of distance to the vent center. This pattern has been frequently observed in shallow-sea hydrothermal systems, such as those located at Kueishantao Island off NE Taiwan [16, 17] and Milos Island in Greece [18], as well as in deep-sea hydrothermal systems located within the 9°N East Pacific Rise (EPR) [18] and Manus Basin off Papua New Guinea [10]. Metagenomic analysis in shallow-sea hydrothermal systems of Kueishantao Island also found that the abundance of carbon fixation genes and sulfur metabolic genes were quite different between vent inside and upside [16]. In a word, thermal and chemical gradients in hydrothermal systems strongly influence the composition and metabolism of microbial communities [1]. The thermal gradients have many effects on microbes, such as DNA stability [19] and enzymatic activity, while the chemical gradient determines the available energy, electron donors, and acceptor sources of chemolithoautotrophs [20].

Under in situ hydrothermal environments, the thermal and chemical gradients are concomitant [15, 21]. Thus, microbial investigation by directly collecting microbial biomass along the mixing gradients [14, 18, 22] may not well distinguish the independent effects of thermal and chemical gradients on microbial composition and function. In addition, it was found that ambient seawater was the dominant source of microbes in the vent plume [23]. Because cold seawater can continually mix with hydrothermal fluids and quickly flow away with the stream, the microbes that are carried from ambient seawater into hydrothermal fluids do not have enough time to adapt to the extreme hydrothermal environment. Thus, the microbial composition and genetic makeup of microbes directly collected from hydrothermal fluids may not well reflect which microbes can survive and what they can do in hydrothermal fluids.

To resolve these primary concerns, we conducted temperature gradient (65 °C, 45 °C, and 30 °C) incubation experiments using hydrothermal fluids from an acidic white vent (pH = 5.6) and an extremely acidic yellow vent (pH = 2.2) in the shallow-sea hydrothermal system near Kueishantao Islet, Taiwan. We identified the active chemolithoautotrophic microbes by adding ^13^C-labeled NaHCO_3_. Multiple controls were established by adding ^12^C/^13^C-NaHCO_3_ and ^14^N/^15^N-NH_4_Cl (^13^C + ^15^N, ^13^C + ^14^N, ^12^C + ^14^N, and ^13^C) to assess the negligible impact of cross-feeding during incubation, and thus these experiments can serve as biological replicates. After the microbial isotopically labeled DNA was obtained, it was subjected to 16S rRNA gene and metagenomic analyses. These analyses revealed the major metabolic functions of active carbon fixation microbes along the temperature gradient in hydrothermal vents with significantly different pH values, as well as the strategies of these chemolithoautotrophs to survive under high temperature and low pH conditions.

## Material and methods

### Study sites, hydrothermal fluids collection, and physicochemical analysis

A white vent (24.83560° N, 121.96339° E) and a yellow vent (24.83455° N, 121.96339° E) are located within 1 km east of Kueishantao Islet off the Northeast coast of Taiwan. In May 2019, a total of 150 L of hydrothermal fluid were collected in situ from the vent mouth for temperature gradient incubation and physicochemical analysis using titanium-made automatic gas-tight hydrothermal samplers (10 L), by scuba divers equipped with a global positioning system. Seawater from the surface (0.5 m), middle (5 m), and bottom (10 m) layers of the reference site (24.83370° N, 121.96212° E), located 160 m away from the white vent, was collected for physicochemical analysis. The geographic location and geochemical characteristics of the two vents and reference site are shown in Fig. S1.

The physicochemical parameters of both fluids and water samples, including temperature, pH, salinity, dissolved oxygen (DO), sulfide (S^2–^), sulfate (SO_4_^2–^), nitrite (NO_2_^–^), nitrate (NO_3_^–^), ammonium (NH_4_^+^), silicate (SiO_3_^2–^), and dissolved inorganic carbon (DIC), were determined either in situ or in the laboratory according to the methods described by Mei et al. [24]. Dissolved methane was measured using gas chromatography with the gas-stripping method [25].

### Dual-labeling SIP incubation experiments

Fluids were collected within 2 h and filtered through 20 μm mesh to remove large particles. The fluids for temperature gradient incubation were then filled into 10 L polycarbonate (PC) bottles, which had been washed with 10% HCl and filtered fluids. To each of the 10 L PC bottle, ^13^C-labeled NaHCO_3_ (Cambridge Isotope Laboratories, Tewksbury, MA, USA) or ^12^C-labeled NaHCO_3_ (Sigma-Aldrich, St Louis, MO, USA), along with either ^15^N- or ^14^N-labeled NH_4_Cl (Sigma-Aldrich, St Louis, MO, USA), was added to achieve a final concentration of 3 mM additional NaHCO_3_ and 50 μM additional NH_4_Cl. The bottles were incubated in dark at 30 °C, 45 °C, and 65 °C for 24 h. The microbes present in the incubated fluids could be either free-living or attached to small particles (< 20 μm) during their growth. To assess the possibility of cross-feeding that heterotrophic microbes assimilated ^13^C- labeled organic matters released by chemolithoautotrophs during incubation, four treatments were set up for each incubation temperature: ^13^C + ^15^N (where C and N refer to substrates NaHCO_3_ and NH_4_Cl, respectively), ^13^C + ^14^N, ^12^C + ^14^N, and ^13^C amendment only. The solely ^15^N-labeled DNA is used to identify active heterotrophic bacteria, and the higher relative abundance of heterotrophic bacteria in the community obtained from ^13^C- and ^15^N-labeled DNA than that obtained from ^15^N-labeled DNA indicates the occurrence of serious cross-feeding. Microbial biomass for the ^13^C content test was collected both at the beginning and after 24 h of incubation using a pre-combusted (4 h at 500 °C) glass fiber filter with 0.3 μm-pore size (Advantec) [26]. The microbial community was collected from 8 L of the incubated fluids after 24 h of incubation by filtering through 0.22-μm PC filters with a suction pressure of < 0.03 MPa. The filters were flash-frozen in liquid nitrogen and stored at − 80 °C until laboratory analysis.

### DNA extraction, CsCl density gradient ultracentrifugation, and quantitative PCR

Microbial DNA from incubation experiments was extracted using the phenol-chloroform-isoamyl alcohol method [27], and DNA concentration was fluorometrically quantified using a Qubit dsDNA Assay Kit (Invitrogen) and Qubit 2.0 Fluorometer (Invitrogen). Cesium chloride (CsCl) density gradient ultracentrifugation and fractionation were performed following published protocols [28, 29]. The abundance of bacterial and archaeal 16S rRNA gene in each fraction was quantified using quantitative polymerase chain reaction (qPCR) with Bac-338f (ACTCCTACGGGAGGCAGCAG) and Bac-518r (ATTACCGCGGCTGCTGG) primers [30] for bacterial quantification, and Arc-344f (ACGGGGYGCAGCAGGCGCGA) and Arc-806r (GGACTACVSGGGTWTCTAAT) [31] for archaeal quantification on a CFX 96™ real-time system (BIO-RAD). Standard curves were constructed using the target DNA fragments of *Escherichia coli* strain P10 and *Nitrosopumilus maritimus* SCM1, respectively. The PCR mixture consisting of 10 μL of SYBR® Premix Ex Taq™ II (TakaRa), 5 μg of bovine serum albumin, 0.5 μM of each primer, and 1 μL of template DNA, was prepared in a total volume of 20 μL. Each reaction mixture was run in triplicate with the following program: initial enzyme activation at 95 °C for 105 s, followed by 40 cycles of 95 °C for 15 s, 55 °C for 30 s, and 72 °C for 30 s. Triplicate non-template reactions were included as negative controls during each run of the program. The qPCR amplification efficiencies ranged from 95 to 100%, with *R*^2^ > 0.99. The specificity of the qPCR reactions was confirmed by analyzing melting curve and agarose gel electrophoresis. To confirm the correctness of ambiguous products, sequencing was performed.

### Bacterial 16S rRNA gene sequence analysis

The distribution curves of bacterial 16S rRNA gene abundance along the CsCl density gradient were analyzed based on the density and bacterial 16S rRNA gene abundance of the CsCl fractions from each sample (Fig. S2). At each temperature, the density of the peak in the single-peaked curves for the ^13^C + ^15^N sample were heavier than that in the ^13^C sample, suggesting that the bacterial communities were mainly composed by autotrophs and that cross-feeding caused by heterotrophic bacteria was negligible. The bacterial 16S rRNA gene copies at YV 65 °C could not be quantified due to the very low bacterial abundance present. For each sample, an equal volume of DNA solution from 1 − 3 continuous ultra-heavy (UH), heavy (H), or light (L) CsCl gradient fractions containing the most abundant bacterial 16S rRNA gene copies (as shown in Fig. S2) were mixed together for high-throughput sequencing to obtain bacterial populations that incorporated both NaH^13^CO_3_ and ^15^NH_4_Cl, only NaH^13^CO_3_, or no labeled substrates. The bacterial V3–V4 hypervariable regions in 16S rRNA genes were amplified using barcode sequences and universal primers Bac-338F (ACTCCTACGGGAGGCAGCA) and Bac-806R (GGACTACHVGGGTWTCTAAT) [32]. The amplicons were then sequenced on an Illumina MiSeq PE300 platform at Guangdong Magigene Biotechnology Co., Ltd. (Guangzhou). The quality-controlled sequences were classified and clustered into operational taxonomic units (OTUs) with a cutoff value of 0.03 using the Mothur software following standard operating procedures (www.mothur.org/wiki/MiSeq_SOP) [29, 33]. To normalize the data, sequences in all samples were rarefied and subsampled to an equal number, which then generated OTU relative abundance matrices for further analysis. The Bray–Curtis dissimilarities between communities were calculated using the OTU relative abundance matrices, after which nonmetric multidimensional scaling ordinations were generated using the vegan package in R. The representative sequences of OTUs were aligned using MEGA7, and phylogenetic trees were constructed using the maximum likelihood method.

### Metagenome sequencing, assembly, and mapping

The UH, H, and L fractions of the samples incubated with NaH^13^CO_3_ and ^15^NH_4_Cl were also subjected to metagenomic sequencing. The sequencing libraries were prepared using NEB Next® Ultra™ DNA Library Prep Kit for Illumina® (New England Biolabs, MA, USA) following the manufacturer’s recommendations. The libraries were then sequenced on an Illumina HiSeq 2500 platform to generate 150 bp paired-end reads. The metagenomic raw reads were trimmed using Trimmomatic v.0.36 with the parameters LEADING: 3, TRAILING: 3, SLIDINGWINDOW: 5:20, and MINLEN: 50. The resulting clean reads were then merged and assembled using MEGAHIT (v.1.0.6, https://github.com/voutcn/megahit) with the following parameters: k-min 35, k-max 95, and k-step 20. The assembled scaffolds that contained one or more continuous N were strictly split from the N connection to produce no-N-contained contigs. Clean reads from each sample were then mapped onto their respective contigs using MEGAHIT (v.1.0.6). The reads that were not mapped onto contigs of all samples were then subject to mixed assembly and interruption as described above to obtain contigs for low-abundance species. Fragments shorter than 500 bp in all of contigs were filtered out for statistical analysis.

### Gene prediction and abundance analysis

For contigs larger than 500 bp, open reading frames (ORFs) were predicted using MetaGeneMark v.3.38. Predicted ORFs that were less than 90 nt in length were filtered out from the final results using the default parameters. The remaining ORFs were then used to generate a non-redundant gene catalog (Unigenes) consisting of unique and continuous nucleotide sequences. The Unigenes were clustered at 95% identity and 90% coverage, with the longest sequence representing each cluster, using CD-HIT v.4.7. To quantitatively compare key genes among the samples, the clean data of each sample was mapped to the Unigenes using BBMAP software (http://jgi.doe.gov/data-and-tools/bbtools/) to determine the number of reads that aligned to each gene in each sample. Based on the number of mapped reads and the length of gene, the normalized relative abundance of each Unigene was determined as Eq. 1:

1 $${G}_{k}=\frac{{r}_{k}}{{L}_{k}}\times \frac{1}{{\sum }_{i=1}^{n}\frac{{r}_{i}}{{L}_{i}}}$$

where ${r}_{k}$ is the number of reads mapped to gene k, ${L}_{k}$ is length of gene *k*.

### Taxonomy prediction and functional annotation

To obtain the taxonomic information of each gene, the Unigenes were blasted to the sequences of bacteria, archaea, viruses, and fungi extracted from the NR database (version 2018–01-02, https://www.ncbi.nlm.nih.gov/) of NCBI using the DIAMOND software v.0.9.9 [34]. The result with e-value ≤ 1 × 10^−10^ was passed to the LCA algorithm using MEGAN to retrieve taxonomic affiliations [35]. Functional annotation was performed by searching against KEGG database (Version 2018–01–01, http://www.kegg.jp/kegg/) and eggNOG database (Version 4.5, http://eggnogdb.embl.de/#/app/home) using Diamond, with only the top hit being retained.

### Metagenomic binning and relative abundance and taxonomy/functional annotation of MAGs

Clean reads from the metagenomes of the UH, H, and L fraction of each sample were merged and assembled using MEGAHIT (v.1.0.6, https://github.com/voutcn/megahit) [36]. The contigs longer than 2500 bp were used to recover metagenomics assembled genomes (MAGs) using MetaBAT2 (v2.12.1, http://bitbucket.org/berkeleylab/metabat) with default parameter [37]. The CheckM (v1.1.3, http://ecogenomics.github.io/CheckM/) was adapted to estimate the completeness and contamination of MAGs [38]. Clean reads of the three metagenomes were aligned to the Scaftigs of each MAG using BBmap (v. 37.36) with default parameters [39]. The relative abundance of each MAG in each metagenomic library was calculated as the proportion of uniquely mapped and correctly paired reads out of the total reads in each metagenome [40].

Taxonomy was assigned to each MAG based on the Genome Taxonomy Database (GTDB, http://gtdb.ecogenomic.org) R05-RS95 using GTDB-Tk v.1.3.0 (https://github.com/Ecogenomics/GtdbTk) [41]. The gene functions of MAGs were annotated by performing a blast search against KEGG databases, using an e-value cutoff of < 1 × 10^−10^ and minimal alignment length larger than 40%.

### Phylogenetic tree construction

According to the taxonomy of the chemolithoautotrophic MAGs, reference genomes were searched in EzBiocloud (https://eztaxon-e.ezbiocloud.net/) [42] and downloaded from NCBI. The phylogenomic trees of the chemolithoautotrophic MAGs and their reference genomes were constructed based on the pipeline of PhyloPhlAn 3.0 by selecting parameters of markers database: phylophlan, diversity: low, and configuration file: supermatrix_aa.cfg [43]. We generated multiple output trees, and the final phylogeny was produced by RAxML staring from the FastTree phylogeny. The resulting tree was visualized using iTOL version 6 (https://itol.embl.de/). The amino acid sequences of the reference genomes were predicted using Rapid Annotation using Subsystem Technology (RAST) [44] and assigned to KO numbers through GhostKOALA [45]. The key genes of the main metabolic pathways and genes related to high temperature and low pH tolerance of the MAGs and reference genomes were shown on the right side of the phylogenomic tree.

### Calculation of DIC uptake rates

The amount of organic carbon and the carbon isotopic compositions (δ^13^C) retained on each glass fiber filter was measured using the combustion method, as described in previous studies [46, 47]. Briefly, the filters were lyophilized for over 16 h and then transferred to disposable petri dishes with a diameter of 47 mm. The filters were then steamed with 1 N HCl for 48 h, dried overnight at 50 °C, and placed into 5 × 9 mm tin cups. The carbon content and δ^13^C values were measured using Elementary analysis-isotope ratio mass spectrometers (EA-IRMS, EA: vario PYRO cube, IRMS: Isoprime 100). International isotope standards USGS 40 (δ^13^C =  − 26.39‰), USGS 41 (δ^13^C = 36.55‰), and IAEA600 (δ^13^C =  − 27.77‰) were used to calibrate the δ^13^C and the analytical precision was within 0.1‰.

The bulk uptake rate of a C source in incubated seawater was calculated using Eqs. 2 − 4:

2 $$\mathrm\it{R}_\text{Sample}=\left(\frac{\delta^{13}C_\text{POC}}{1000}+1\right)\times{R_{VPDB}}$$

3 $$\text{n=}\frac{{\textit{R}}_{\text{sample}}}{{\textit{R}}_{\text{sample}}+ {1} }$$

4 $$Assimilation\,rate_\text{DIC}=\frac{POC_\mathrm{t}\times{n_\mathrm{t}}-POC_0\times{n_0}}{t}\times\,\frac{Ca_\text{DIC}+Cs_\text{DIC}}{Cs_\text{DIC}}$$

where ${\textit{R}}_{\text{sample}}$ is the ratio of ^13^C/^12^C, $\it {\text{R}}_{VPDB}$ is the atomic percent of ^13^C in the international reference material Vienna Peedee Belemnite (VPDB), and its value is 0.0112372 [48], ${\textit{n}}_{\text{t}}$ and ${\textit{n}}_{0}$ are the atomic percentages of ^13^C-particle organic carbon (POC) at the end and beginning of an incubation, ${\textit{POC}}_{\text{t}}$ and ${\textit{POC}}_{0}$ are the POC concentrations at the end and beginning of an incubation, ${\textit{Ca}}_{\text{DIC}}$ and ${\textit{Cs}}_{\text{DIC}}$ are the ambient and added DIC concentrations, respectively, and *t* is the incubation time.

## Results

### Biogeochemical parameters

**Fig. 1:**
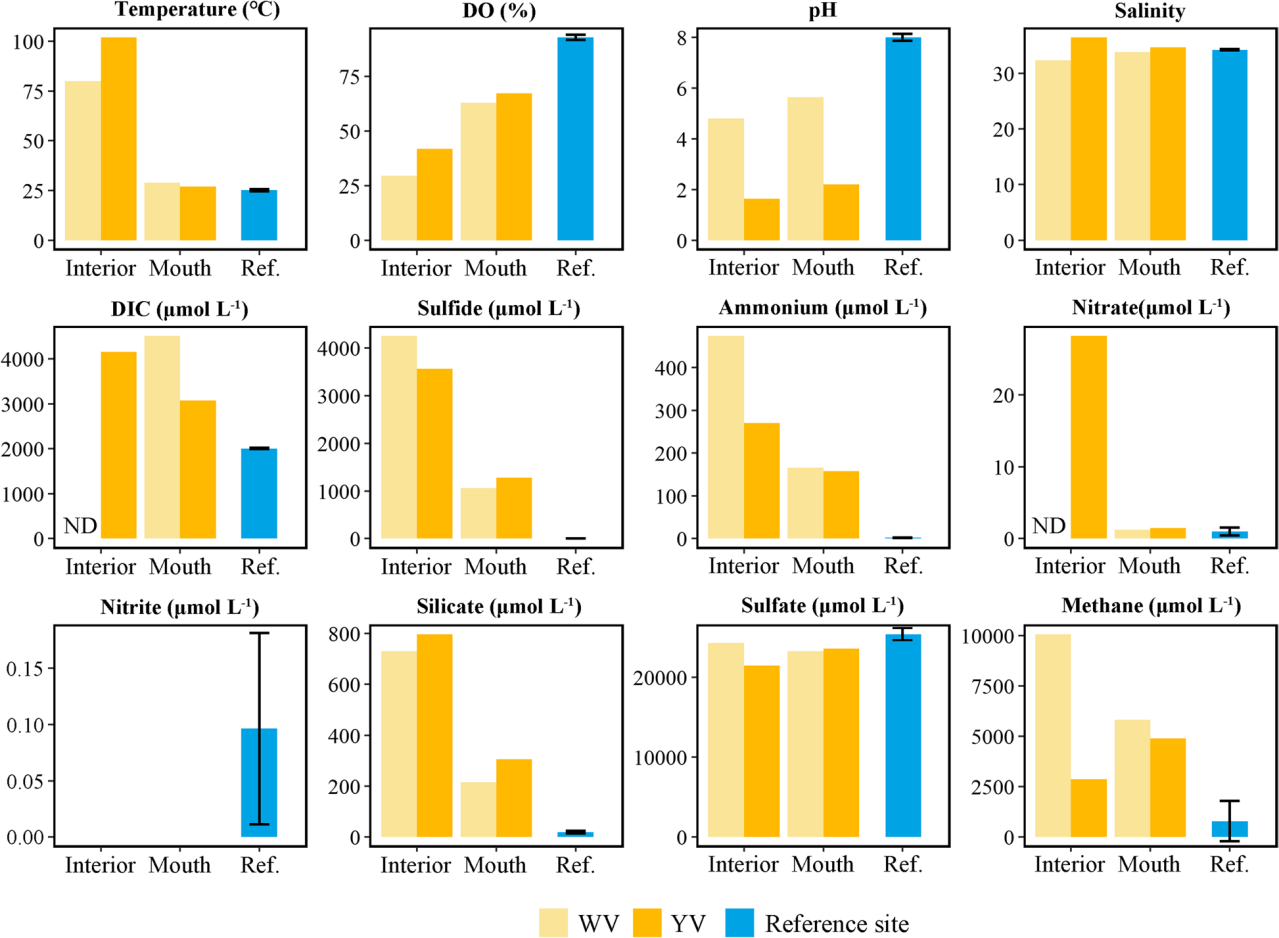
Physicochemical parameters of the white vent (WV), yellow vent (YV), and reference site. The error bars represent the standard deviation of the mean of measurements taken from three depth layers at the reference site. DO, dissolved oxygen; DIC, dissolved inorganic carbon; DOC, dissolved organic carbon; ND, no data

The physicochemical parameters were quite different between the vent interior and the reference site (Fig. 1). The white vent (WV) and yellow vent (YV) were located at depths of 13.9 and 8.5 m on the seafloor, with interior temperatures of 80 and 102 °C, respectively. The pH inside YV (1.63) was much lower than that inside WV (4.81), but both were much lower than the pH at the reference sites (7.85 − 8.08). The dissolved oxygen inside both vents (29.9 − 41.9%) were lower than those at the reference sites (91.5 − 93.8%). S^2−^ was more than 3500 μmol L^−1^ inside the two vents, but was not detected at the reference sites. In addition to S^2−^, the concentration of other reduced matter, including NH_4_^+^ and CH_4_, was also much higher inside the two vents than at reference sites. This indicates that the Kueishantao shallow-sea hydrothermal system had abundance of reduced matters to support the energy requirements of chemolithoautotrophs. The concentrations of dissolved inorganic carbon (DIC) inside the two vents (3073 − 4514 μmol L^−1^) were more than 1.5 times higher than those at the reference sites. The NO_3_^−^ concentration inside the vent (28.2 μmol L^−1^) was much higher than at reference sites (0.63 − 1.6 μmol L^−1^), which suggests that the NO_3_^−^ released from the vent interior to seawater might be quickly consumed by microorganisms. After the hydrothermal fluids from the vent interiors were mixed with seawater at the vent mouths, where the fluids were collected for temperature gradient incubation, the values of the physicochemical parameters were located between the values of those inside the vents and at the reference sites. The most notable difference between them was that the pH at YV mouth (pH = 2.2) was much lower than at WV mouth (pH = 5.6).

### Prokaryotic abundance and carbon fixation rate in the vent mouth

Under in situ conditions, bacterial 16S rRNA gene abundance at WV and YV mouth were 5.51 × 10^5^ copies mL^−1^ and 1.34 × 10^4^ copies mL^−1^, respectively, while archaeal 16S rRNA gene abundance was 6.36 × 10^2^ copies mL^−1^ at WV mouth and undetectable at YV mouth (Fig. S3). This indicates that the prokaryotic community at the vent mouth was mainly comprised by bacteria [11]. After incubation, the addition of isotopic substrates under each temperature had no significant effect on bacteria abundance (Kruskal–Wallis test, *P* > 0.05) (Fig. S3). The bacterial abundances in the WV samples slightly changed with the change of temperature (Kruskal–Wallis test, *P* > 0.05), but in the YV samples, they significantly decreased when the temperature was rising from 30 to 65 °C (Kruskal–Wallis test, *P* < 0.01) (Fig. S3). The archaeal abundances in the WV samples showed an increase with the increasing temperature (Kruskal–Wallis test, *P* < 0.05), while they were only detected in one sample at YV 45 °C and in three samples at YV 30 °C (Fig. S3).

The carbon fixation rates were calculated based on the δ^13^C of biomass collected at the end of incubation (Table 1). Similarly, the addition of NH_4_^+^ did not affect the carbon fixation rate (one-way ANOVA, *P* > 0.05). In WV (pH = 5.6), the average carbon fixation rate of the three ^13^C added samples that incubated at 65, 45, and 30 °C was 1.5 ± 0.12, 2.06 ± 0.29, 1.78 ± 0.17 μmol C L^−1^ day^−1^, respectively. The carbon fixation rates from YV (pH = 2.2) samples were much lower than WV samples (Wilcoxon rank-sum test, *P* < 0.01), and decreased from 0.063 ± 0.0018 μmol L^−1^ day^−1^ at 30 °C to undetectable levels at 65 °C. Consequently, the carbon fixation rates in Kueishantao shallow-sea hydrothermal vents were within the range of values reported from previous studies in hydrothermal fluids [4, 49, 50], and were not significantly affected by the temperature of 30 − 65 °C under the condition of pH = 5.6. However, they could be significantly restrained with the increase of temperature when the pH is lower (pH = 2.2).

**Table 1:** Carbon fixation rate calculated based on the ^13^C content of microbial biomass

Site	Temperature	Carbon fixation rate (μmol L^−1^ day^−1^)			
		^13^C + ^15^N	^13^C + ^14^N	^13^C	Average
WV	65 °C	1.43	1.68	1.38	1.50 ± 0.12
	45 °C	1.82	2.49	1.87	2.06 ± 0.29
	30 °C	1.67	2.04	1.63	1.78 ± 0.17
YV	65 °C	BDL	BDL	BDL	ND
	45 °C	0.017	0.012	0.013	0.014 ± 0.002
	30 °C	0.054	0.09	0.046	0.063 ± 0.0018

*BDL* Below detection limit. *ND* No data, ^13^C represents substrate NaH^13^CO_3,_ ^15^N and ^14^N represent substrate ^15^NH_4_Cl and ^14^NH_4_Cl, respectively

### Active carbon fixation taxa along the temperature and pH gradient

**Fig. 2:**
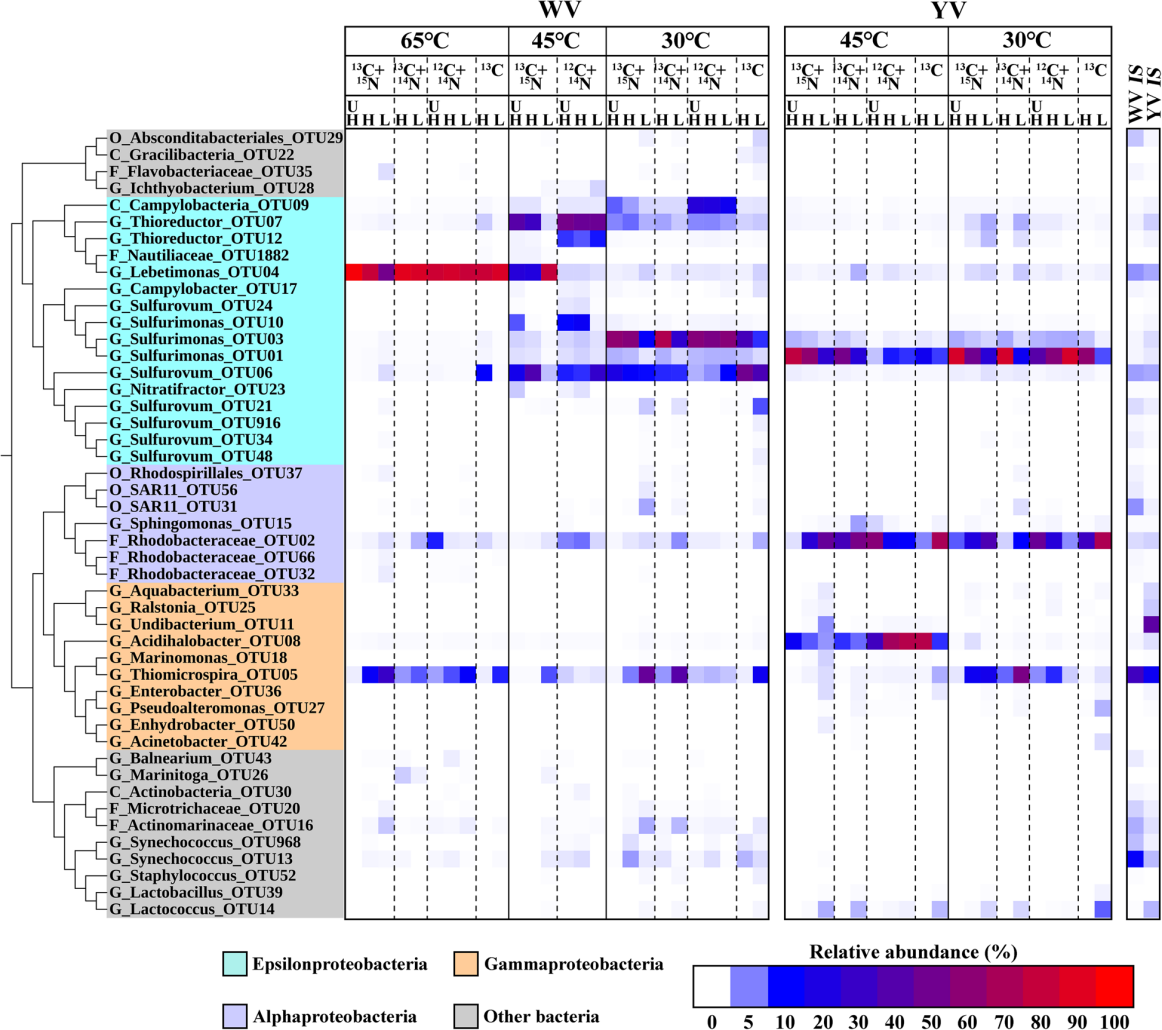
Phylogenetic tree of bacterial operational taxonomic unit (OTU) sequences with a relative abundance > 0.5% of the total 16S rRNA gene sequences in at least one of the representative ultraheavy, heavy, and light fractions. The relative abundances are shown as heat maps to the right of the phylogenetic tree. The figure was produced from the Interactive Tree Of Life (iTOL, http://itol.embl.de/). ^13^C and ^12^C represent substrate NaH^13^CO_3_ and NaH^12^CO_3_, ^15^N and ^14^N represent substrate ^15^NH_4_Cl and ^14^NH_4_Cl, respectively. WV, white vent; YV, yellow vent; *IS*, in situ; C, class; O, order; F, family; G, genus

The amplicon sequencing was carried out only for bacterial communities since they constituted more than 99.8% of the total prokaryotic community (Fig. S3). The DNA from CsCl density fractions of samples at YV 65 °C were not sequenced (Fig. 2) due to the much lower bacterial abundance (Fig. S3). The bacterial community compositions in the WV 45 °C and 30 °C samples were more similar to each other, but they were more different from the community composition in the WV 65 °C sample (Fig. S4a). In addition, bacterial community compositions between WV and YV were quite different (Fig. S4a). These results indicate the high temperature (65 °C) and low pH had a significant effect on the bacterial community composition.

**Fig. 3:**
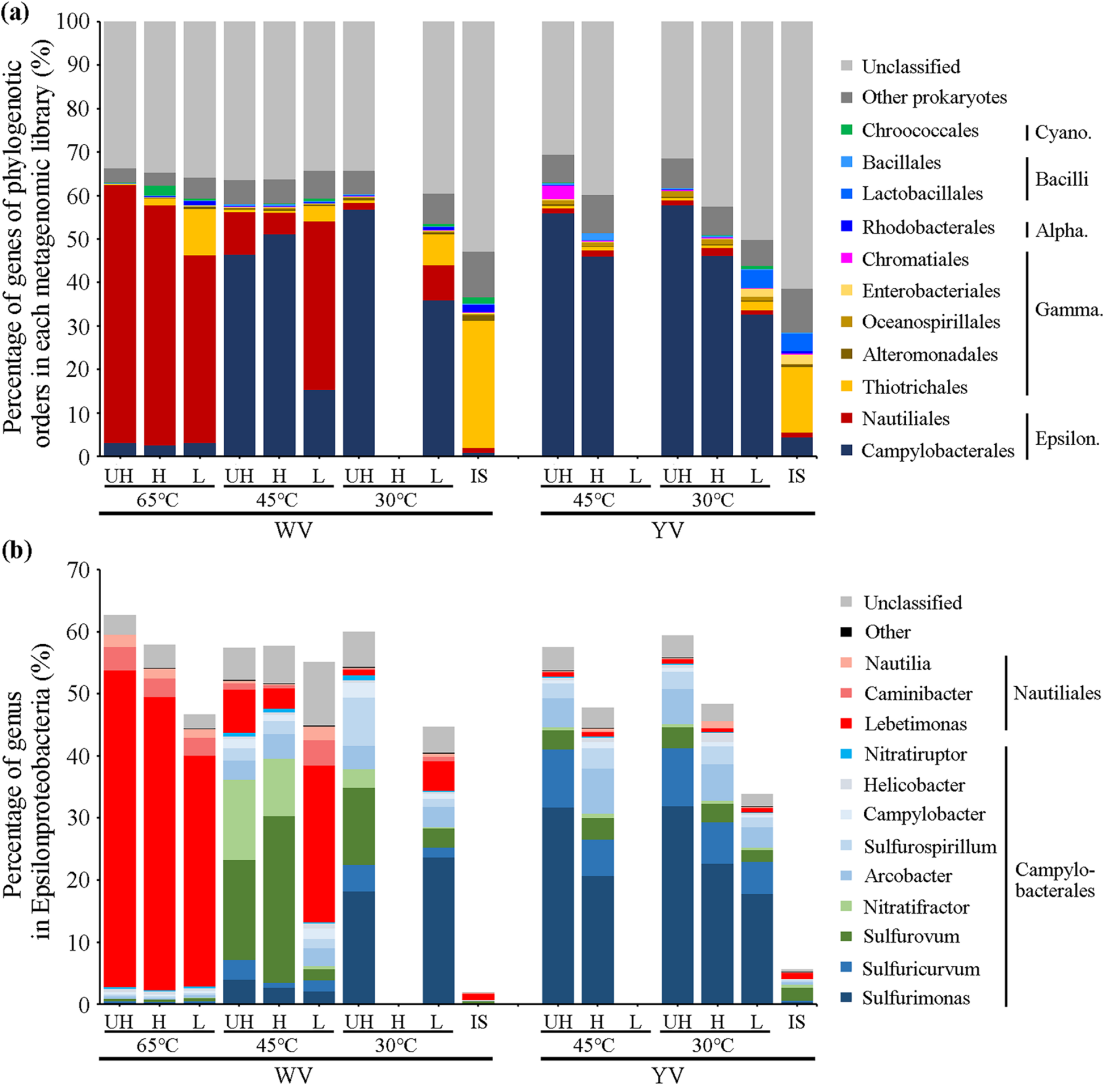
Relative abundance of reads assigned to phylogenetic (**a**) orders and (**b**) genus of *Epsilonproteobacteria* based on metagenomic data

In DNA-SIP analysis, for the ^13^C substrate amended sample, if a taxon actively incorporated ^13^C substrate, its relative abundance in heavy fraction would be higher than in light fraction or similar between heavy and light fraction; if it did not incorporate ^13^C, its relative abundance would decrease significantly from light fraction to heavy fraction [48, 51]. Taxonomic analysis based on the 16S rRNA gene showed that the carbon fixation activity of *Lebetimonas* (order *Nautiliales* in *Epsilonproteobacteria*) was highest at WV 65 °C and reduced with the decrease in temperature (Fig. 2). *Thioreductor*, another member of *Nautiliales*, showed the highest carbon fixation activity at WV 45 °C. In contrast, *Sulfurovum* and *Sulfurimonas* (order *Campylobacterales* of *Epsilonproteobacteria*) were found to actively fix carbon at 45 and 30 °C in the two vents. However, different OTUs of *Sulfurimonas* were abundant in the WV 45 °C, WV 30 °C, and YV 45 °C and 30 °C samples, respectively. *Thiomicrospira* (order *Thiotrichales* in *Gammaproteobacteria*) was the most abundant taxon in in situ environment of the two vent mouths but showed no carbon fixation activity in any of the incubated samples (Fig. 2). *Acidihalobacter* (order *Chromatiales* in *Gammaproteobacteria*), which is a halotolerant acidophile that was first isolated from a geothermally heated seafloor [52, 53], showed carbon fixation activity at YV 45 °C. *Rhodobacteraceae* was the only chemoheterotroph widely existing in all incubated samples and showed no carbon fixation activity. The relative abundance profiles of *Nautiliales*, *Campylobacterales*, *Thiotrichales*, and *Chromatiales* among these samples were similar between metagenomic and 16S rRNA-based data (Figs. 3a and S5). A small part of *Synechococcus* (up to 3%) was detected in the WV samples in both 16S rRNA gene and metagenomic datasets and slightly enriched in the H fraction (Figs. 2 and 3). A previous microbial investigation of seawater around the vent of the Kueishantao hydrothermal system, based on metatranscriptome analysis, has detected the activity of *Synechococcus* in the vent ambient seawater [11].

### Distribution of key metabolic genes along temperature and pH gradient

**Fig. 4:**
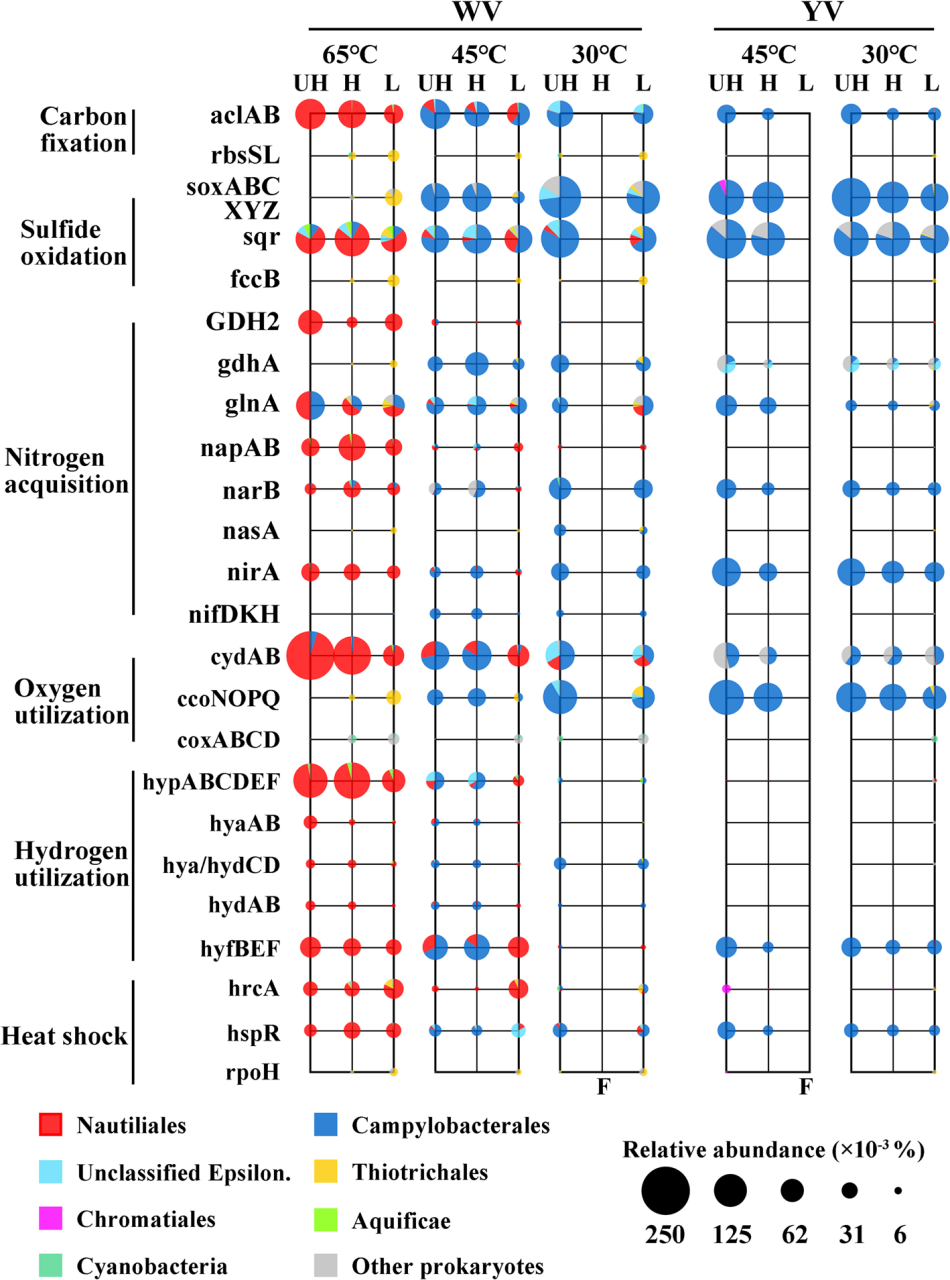
Relative abundance and taxonomic composition of reads annotated to the key genes of carbon fixation, sulfide oxidation, nitrogen acquisition, oxygen utilization, hydrogen utilization, and heat-shock protein regulator in the ultra-heavy (UH), heavy (H) and light (L) metagenomes from each incubated sample. Unclassified reads at the kingdom level were excluded from statistical analysis

Temperature and pH significantly affected not only the bacterial community structure (Fig. S4a) but also their functional composition (Fig. S4b). Key genes involved in carbon fixation, sulfur oxidation, nitrogen acquisition, oxygen and hydrogen utilization, and heat-shock regulation were examined (Fig. 4) to assess the major metabolic functions of autotrophs under different temperature and pH conditions.

#### Genes related to carbon fixation and energy acquisition

Genes encoding for the ATP-citrate lyase (*aclA/B*), which is the key enzyme of the rTCA cycle, were most abundant in all UH fractions, and most of them shared high similarities with those from *Nautiliales* and *Campylobacterales* (Fig. 4). Additionally, a small percentage (0.06 to 2.54%) of *aclA/B* were assigned to *Aquificae*, but these sequences were consistently absent in all UH fractions. Genes encoding for the key enzyme of the Calvin-Benson-Bassham (CBB) cycle, ribulose-1,5-bisphosphate carboxylase (*rbsL/S*), were assigned to *Thiotrichales* and were found to be much more abundant in the L fraction than in the UH or H fraction in all samples. Meanwhile, those genes assigned to *Chromatiales* were most abundant in the UH fraction of both YV 45 °C and 30 °C samples (Fig. 4).

The gene encoding for the sulfide-quinone oxidoreductase (*sqr*), which is involved in sulfur oxidation, was abundant (0.08 − 0.15%) across all samples, and it was found to be present in all chemolithoautotrophic populations (Fig. 4). In contrast, genes encoding for thiosulfate oxidation multienzyme complex (*soxA/B/C/X/Y/Z*) were assigned *Campylobacterales*, *Thiotrichales*, and *Chromatiales*, but not assigned to *Nautiliales*. Therefore, these genes were detected to be abundant only under 45 °C and 30 °C (0.09 − 0.2%), but almost undetectable in the UH fraction of the WV 65 °C sample (Fig. 4). The gene encoding for flavocytochrome c-sulfide dehydrogenase flavoprotein chain (*fccB*) was found to share high similarity only with those from *Thiotrichales*.

Genes encoding for [Ni–Fe] hydrogenase group1 (Hyd1) small subunit (*hyaA*) and large subunit (*hyaB*), as well as quinone-reactive [Ni/Fe]-hydrogenase (Hyd5) small subunit (*hydA*) and large subunit (*hydB*), were mainly detected at WV 65 °C and 45 °C (0.009% − 0.03%) for hydrogen utilization (Fig. 4). Genes encoding for the Hyd1 or Hyd5 cytochrome *b* subunit *(hya/hydC*) and hydrogenase maturation protease (*hya/hydD*) were found to be similarly abundant as *hyaAB* and *hydAB* at WV 65 °C and 45 °C, and they were also abundant at WV 30 °C. Similarly, the genes encoding for the proteins that are involved in the maturation of [Ni–Fe] hydrogenases (*hypA/B/C/D/E/F*) were mainly detected at WV 65 °C and 45 °C, and they were more abundant than the *hya* and *hyd* genes, particularly at WV 65 °C. Unlike Hyd1 and Hyd5, genes encoding for the B/E/F subunits of the [Ni–Fe] hydrogenase group4 (Hyd4) (*hyfB/E/F*) were identified to be abundant both at WV 65 °C and 45 °C, as well as at YV (0.04 − 0.07%). Most of these genes were assigned to *Nautiliales* and *Campylobacterales*, and they were most abundant in the UH fraction of all samples.

#### Nitrogen acquisition

The gene encoding for NAD(H)-linked glutamate dehydrogenase (*GDH2*), which is involved in ammonium assimilation, was primarily detected at 65 °C and assigned to both *Nautiliales* (which were dominant at 65 °C) and *Campylobacterales* (which were dominant at 30 and 45 °C). In contrast, the gene encoding for the NADP(H)-linked glutamate dehydrogenase (*gdhA*) was mainly assigned to *Campylobacterales* and *Thiotrichales*, and thus was only abundant at 45 and 30 °C (Fig. 4). The gene encoding for GS (*glnA*), which is the first enzyme of the glutamine synthetase and glutamate synthase (GS-GOGAT) pathway of ammonium assimilation, was found to be abundant (0.01 − 0.09%) across all samples.

Like *GDH2*, genes encoding for the periplasmic nitrate reductase (*napA/B*), which are involved in dissimilatory nitrate reduction, were assigned to both *Nautiliales* and *Campylobacterales*, but were only detected to be abundant at WV 65 °C (Fig. 4). For the assimilatory nitrate reduction pathway, genes encoding for the ferredoxin-nitrate reductase (*narB*) and ferredoxin-nitrite reductase (*nirA*) were abundant in all samples (~ 0.01%). The gene encoding for the assimilatory nitrate reductase catalytic subunit (*nasA*) was only abundant at WV 30 °C. Genes encoding for subunits of the nitrogenase (*nifD/K/H*) were mainly identified at WV 45 °C and 30 °C (~ 0.01%), with more than 98% of them assigned to *Campylobacterales*.

#### Oxygen utilization

Genes encoding for the subunits of cytochrome bd ubiquinol oxidase (*cydA/B*) were the most abundant genes at WV 65 °C (Fig. 4), and they were significantly enriched in the UH fractions of the WV 65 °C sample (0.3%) compared to the WV 45 and 30 °C samples (0.04 − 0.1%) (Fig. 4). However, genes encoding for the subunits of cytochrome c oxidase cbb3-type (*ccoN/O/P/Q*) were mainly assigned to *Campylobacterales* and *Thiotrichales*. Only those assigned to *Campylobacterales* were found to be abundant at 45 and 30 °C (0.03 − 0.1%). Genes encoding for subunits of cytochrome c oxidase aa3-type (*coxA/B/C/D*), which are typically expressed in aerobic conditions, were found to be most abundant in the L fraction across all samples.

### Impact of temperature and pH on metagenome compositions

**Fig. 5:**
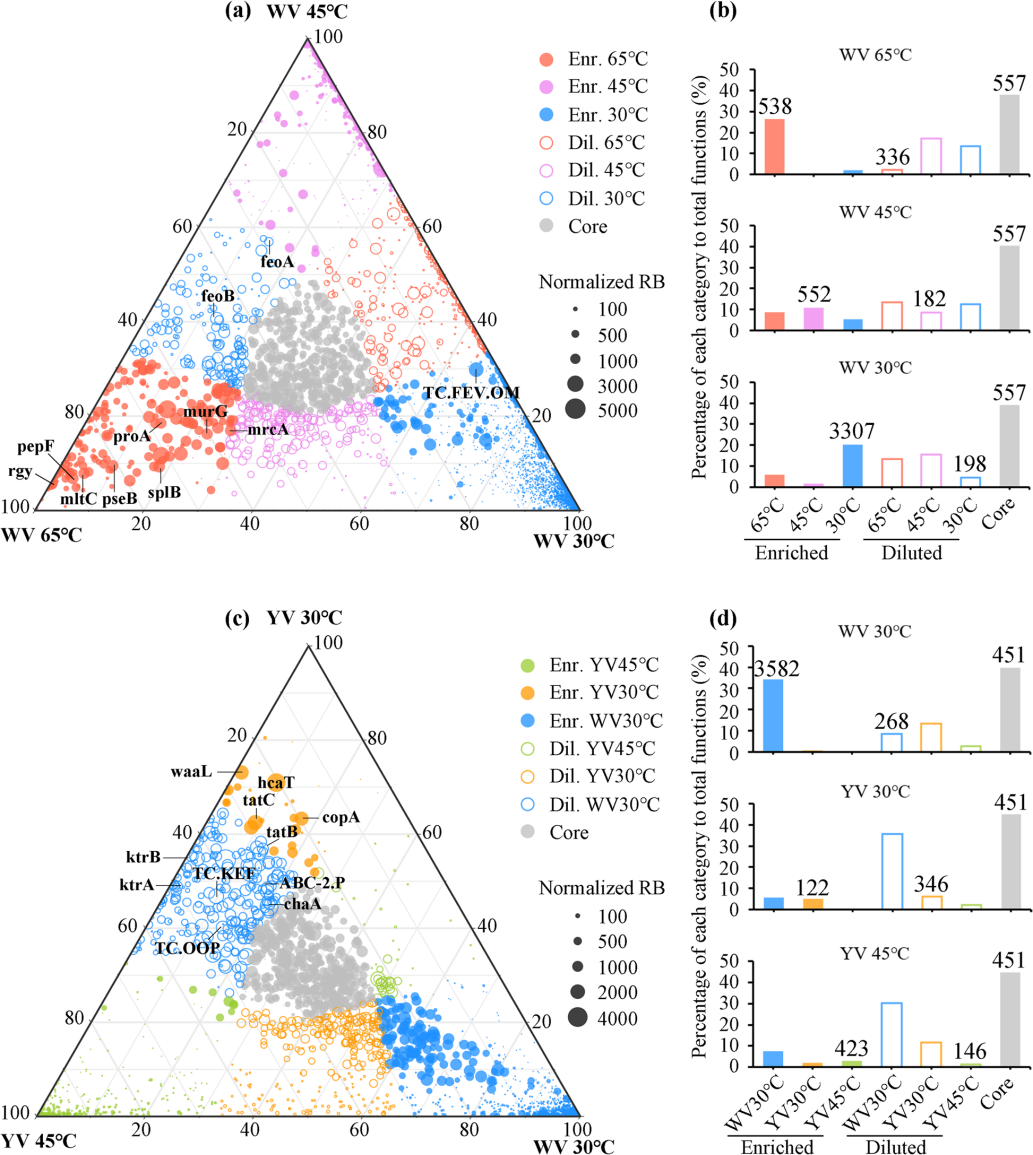
Comparison of KEGG Orthologys (KOs) (**a,b**) among 65, 45, and 30 °C at site WV, and (**c,d**) among 30 °C at site WV and 30 and 45 °C at site YV. In **a** and **c**, circle size represents the highest normalized relative abundance (RB) of each KO among the three metagenomic libraries. If the difference in relative abundance of a KO was less than two-fold across the three metagenomic libraries, it was noted as a core function (gray circle). If the relative abundance of a KO in one metagenomic library was at least twofold higher than its abundance in the remaining two libraries, the KO was noted as an enriched (Enr.) function (closed color circles) in that library. Functions with lower relative abundance in one metagenomic library compared to the other two libraries were classified as “diluted (Dil)” KOs (open circles) in that library. In **b** and **d**, the total relative abundance of each KO category is shown, with the number of KOs in each category indicated above the bars

Based on the analyses of Fig. 4, gene composition in UH-DNA of each sample can best reflect the metabolic functions of active chemolithoautotrophs. Therefore, we conducted a metagenomic comparison of UH fraction from the three temperatures at WV to further access the survival strategies of chemolithoautotrophs under high temperature (Fig. 5a,b). In total, 5490 KEGG Orthologs (KOs) were identified from WV 65, 45, and 30 °C (Fig. 5a). Out of those, 557 KOs were identified as core functions, constituting a total relative abundance that ranged from 38 to 40.5% across the three temperatures (Fig. 5b). For enriched KOs at each temperature, the number of KOs enriched at 65 °C was the least, but they had the highest in relative abundance (Fig. 5a,b). On the contrary, there were a lot of KOs (located near or on the axis of 30 − 45 °C) with low abundance at 30 and 45 °C that were absent or almost absent at 65 °C (Fig. 5a).

As the microbial community in WV samples at 30 °C and YV samples at 45 and 30 °C were mainly composed of *Sulfurimonas* but different OTUs (Figs. 2 and 3), the composition of KOs among the metagenomes of the UH fractions from those samples was compared (Fig. 5c,d) to assess the strategies they used to inhabit under low pH. Among the 5340 KOs identified from the three metagenomes, the total relative abundance of 451 core KOs ranged from 39.8 to 45% (Fig. 5d). The total relative abundance of the 3582 KOs enriched at WV 30 °C was 34.2% at WV 30 °C, whereas only a small portion of KOs enriched at YV 30 and 45 °C. Instead, only 268 KOs were shared by microbes at YV 30 and 45 °C (Dil. WV 30 °C in Fig. 5d), which accounted for more than 30% of total KOs abundance at YV 30 and 45 °C, respectively (Fig. 5d). The comparison profile of KOs among WV 45 °C, YV 45 °C, and YV 30 °C (Fig. S6) was similar with those among WV 30 °C, YV 45 °C, and YV 30 °C (Fig. 5c,d). These results suggest that the microbial community, mainly comprised of *Epsilonproteobacteria*, utilized a stable proportion of essential functions (KOs) to support their fundamental metabolic activities across temperature and pH gradients. Additionally, they frequently relied on a small subset of specific KOs to ensure survival under high temperature and low pH conditions. Compared to the core functions pool (Fig. S7), the proportion of amino acid metabolism, metabolism of cofactors and vitamins, and nucleotide metabolism were largely decreased in the enriched function pool. On the other hand, member transport, signal transduction, and unclassified and unknown functions were substantially increased in the enriched function pool (Fig. S8).

Among the enriched KOs with a relative abundance > 1000 per million at WV 65 °C, the genes encoding for the enzymes that have been proven essential or may play important roles in microbial growth under high temperature include reverse gyrase (*rgy*, K0170), glutamate-5-semialdehyde dehydrogenase (*proA*, K00147), spore photoproduct lyase gene (*splB*, K03716), oligoendopeptidase F (*pepF*, K08602), *mrcA* (K05366), murG (K02563), and *mltC* (K08306) (Fig. 5a). Among the KOs with relative abundances more than two times higher at YV 45 °C and YV 30 °C than at WV 30 °C, and with a relative abundance > 1000 per million at YV 45 °C or YV 30 °C, the KOs that may participate in the pH homeostasis of cells include genes encoding for monovalent cation/H^+^ antiporter (*TC.KEF*, K03455), Ca_2_^+^/H^+^ antiporter (*chaA*, K07300), trk system K^+^ uptake protein (*ktrB*, K03498; *ktrA*, K03499), Cu^2+^ exporting ATPase (*copA*, K17686), ABC-2 type transport system permease protein *ABC-2.P* (K01992), O-antigen ligase (*waaL*, K2847), and porin *TC.OOP* (K03286) (Fig. 5c).

### Comparative analyses of metagenomics assembled genomes (MAGs) and genomes

**Fig. 6:**
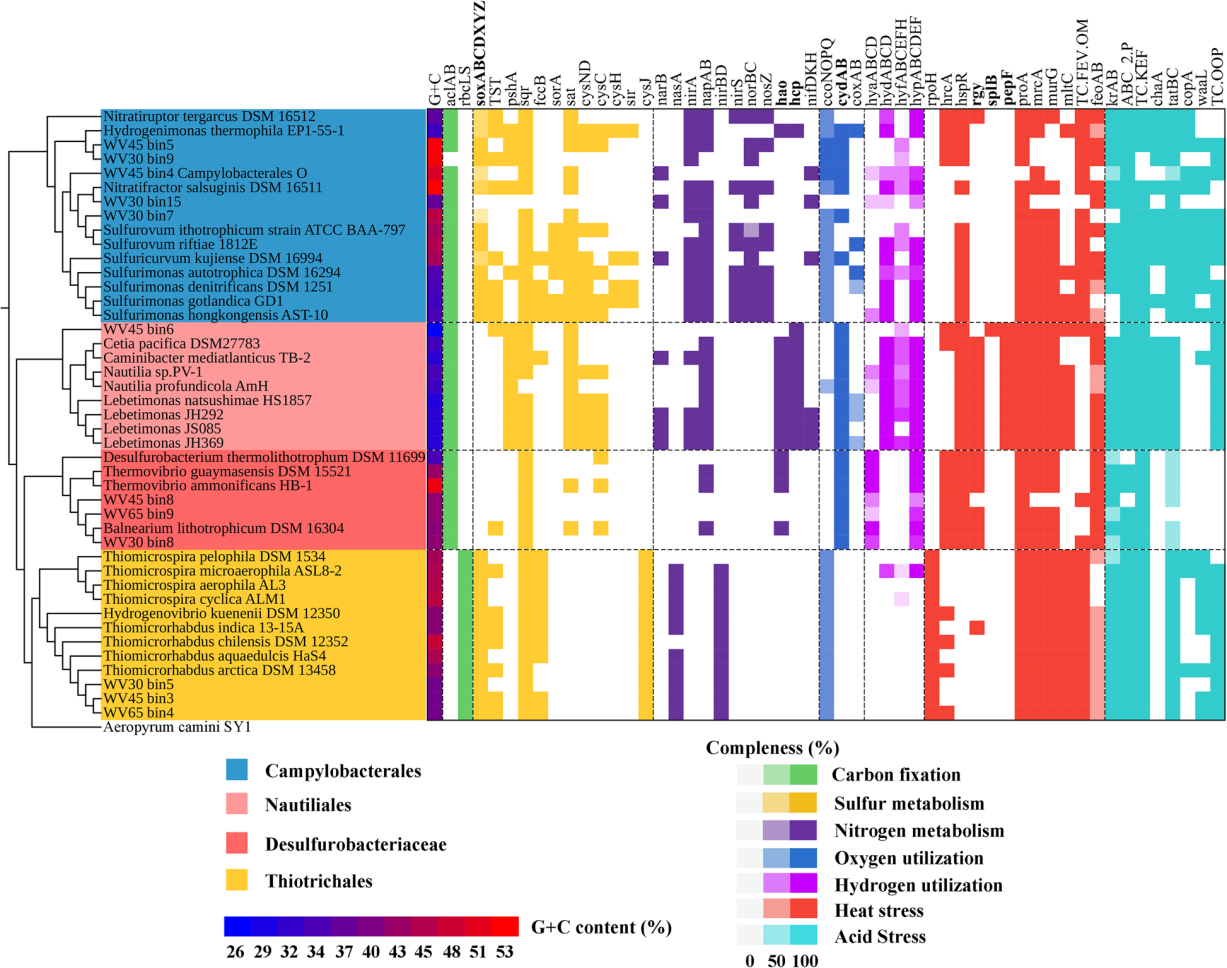
Phylogenetic tree of metagenome assembled genomes from the present study and their reference genomes. The G + C content and presence of the key genes related to carbon fixation, sulfur and nitrogen metabolism, oxygen and hydrogen utilization, and high temperature and acid tolerance are shown as heat maps to the right of the phylogenetic tree. The figure was produced from the Interactive Tree Of Life (iTOL, http://itol.embl.de/)

After filtration of low-quality MAGs, the high-quality MAGs of chemolithoautotrophs share high similarity with the genomes of *Sulfurovum*, *Nitratifractor*, *Hydrogenimonas*, and unclassified *Campylobacterales*, unclassified *Nautiliales*, *Thiomicrospira*, and *Thermovibrio* (order *Desulfurobacteriaceae* in *Aquificae*) (Table S1). The relative abundance distribution of each MAG along the CsCl density fractions provided evidence for the carbon fixation activity of the members of *Campylobacterales* and *Nautiliales*, while also indicating the inactivity of *Thiomicrospira* and *Thermovibrio* in our study (Table S1). Phylogenetic analyses of the MAGs and their reference genomes revealed that they clustered into four branches: *Nautiliales*, *Campylobacterales*, *Thiotrichales*, and *Desulfurobacteriaceae*. Among these, WV45°C bin6 and WV45°C bin4 appear to belong to an unclassified family in *Nautiliales* and *Campylobacterales*, respectively, within the Kueishantao shallow-sea hydrothermal ecosystem (Fig. 6). Comparative genomic analysis found that *Nautiliales*, *Campylobacterales*, *Thiotrichales*, and *Desulfurobacteriaceae* showed significant differences in gene composition in the terms of sulfur and nitrogen metabolism, hydrogen and oxygen utilization, as well as heat and acid stress tolerance (Fig. 6).

## Discussion

The taxonomy and metabolic capabilities of chemolithoautotrophs inhabiting hydrothermal sulfide chimneys are largely influenced by the local geochemical conditions, particularly temperature and pH [7, 11]. Members in order *Nautiliales*, *Campylobacterales*, and *Thiotrichales* have frequently been found to be the major active bacterial groups in the hydrothermal systems of Kueishantao Island [3, 11, 22]. Our work for the first time showed that *Nautiliales* exhibited high carbon fixation activity at high temperature (65 °C) and moderate acidity (pH = 5.6) conditions, and *Campylobacterales* were adapted to moderate temperature (45 − 30 °C) and moderate and extreme acidity (pH = 2.2) conditions in the hydrothermal systems of Kueishantao Island by using DNA-SIP analysis. However, the *Thiotrichales* did not show carbon fixation activities in any of the samples. In addition, we found that extremely acidic condition (specifically at pH 2.2) restrained the high-temperature tolerances of *Nautiliales*. A previous study has shown that high-temperature tolerances of hyperthermophilic archaea were not greatly affected by pH within the range of 4.5–7.5 [54]. In the present study, we found the archaeal abundance was stimulated under moderately acidic conditions (pH = 5.6), but inhibited under extremely acidic conditions (pH = 2.2) at high temperatures (Fig. S3). These results indicate that varying acidic conditions have distinct impacts on chemolithoautotrophs at different temperatures. Here, DNA-SIP combined with metagenomic analysis provides genomic insights into the impact of temperature and pH on the metabolic functions of the primary chemolithoautotrophs living in the hydrothermal ecosystem.

### High temperature and low pH-induced difference in microbial metabolism

Previous studies conducted in sulfur-rich hydrothermal ecosystems have found that chemolithoautotrophs typically utilize reduced sulfur and H_2_ as energy sources, and inorganic nitrogen as electron acceptors and nitrogen sources to reduce CO_2_ to organic carbon [11, 55]. The transcriptional activities of the *Epsilonproteobacteria* and *Aquificae* rTCA pathways, as well as the *Gammaproteobacteria* CBB pathway for carbon fixation, were frequently detected in marine and terrestial hydrothermal ecosystems [11, 56–59]. Our study found that the rTCA carbon fixation pathway was active in all of our incubation conditions, whereas the *Thiotrichales* CBB pathway was inactive. The *Chromatiales* and *Cyanobacteria* CBB pathways were active at YV and WV, respectively (Fig. 4). These results suggest that temperature and pH might not be the determining factors in the activity of rTCA and CBB cycles.

Given the high concentration of S^2−^ observed in WV and YV (Fig. 1), sulfur oxidation may be a primary energy source for carbon fixation mediated by chemolithoautotrophs in these regions. Our study detected several sulfur-oxidizing genes, including *soxABCXYZ*, *sqr*, and *fccB*. Among these, *sqr* was the only gene that was abundant at WV 65 °C (Fig. 4). SQR is an enzyme frequently observed in hyperthermophiles. For example, the SQR isolated from thermoacidophilic *Acidianus ambivalens* demonstrated maximum activity at 70 °C and was almost inactive at room temperature (25 °C) [60]. The *Campylobacterales* group contained both *sox* genes and *sqr*, while *Nautiliales* only contained *sqr* (Figs. 4 and 6). Notably, we found that all *Nautiliales* and hyperthermophilic *Aquificae* genomes (Table S2) lacked *sox* genes but contained *sqr*. According to many scientific proposals [61, 62], life on Earth may have originated from high-temperature hydrothermal vents, and SQR is considered a phylogenetically ancient enzyme that was acquired early in the evolution of life [63]. The absence of *sox* genes in thermophilic or mesophilic *Nautiliales* may be due to the limited availability of thiosulfate under 65 °C, as thiosulfate can easily hydrolyze into sulfur and sulfur dioxide under acidic condition when the temperature exceeds 45 °C. Thus, it is possible that temperature played an important role in the acquisition of *sox* genes by chemolithoautotrophs during their evolution to adapt to lower temperatures from their high-temperature environments. Although Fcc provides less energy through sulfide oxidation than SQR [64], it has a higher affinity for sulfide [65].

Hydrogen is another important reducing agent present in hydrothermal systems, and its oxidation can yield higher catabolic energy than sulfur oxidation [5]. Therefore, hydrogen was also a significant energy source for chemolithoautotrophs inhabiting hydrothermal vents [11, 55]. In the present study, we found that *Epsilonproteobacteria* exhibit a higher genetic potential for increased activity of Hyd1 and Hyd5, responsible for hydrogen oxidation, under high temperature of 65 °C. However, this potential is inhibited by extremely acidic conditions [66, 67]. Unlike Hyd1 and Hyd5, Hyd4 catalyzes the production of H_2_ depending on electrochemical proton gradient (Δμ_H_^+^) [68–70], of which the membrane subunits HyfDEF are involved in proton-translocating [71]. The high abundance of *hyfBEF* (mainly *hyfEF*) genes in the WV 65 °C and 45 °C samples, as well as in the YV samples (Fig. 4), suggests that *Nautiliales* and *Campylobacterales* could use HyfEF for proton translocation to adapt to acidic environments.

Carbon fixation requires the coupling of nitrogen assimilation with growth [72]. *Nautiliales* and *Campylobacterales* have the potential to utilize both GDH and GS-GOGAT pathway for NH_4_^+^ assimilation (Fig. 4). In the GDH pathway of ammonium assimilation, *Nautiliales* utilize NAD(H)-GDHs while *Campylobacterales* utilize NADP(H)-GDHs (Fig. 4). NADP(H)-GDHs are typically involved in ammonia assimilation [73], whereas NAD(H)-GDHs can generate 2-oxoglutarate from glutamate, an important intermediate in the rTCA cycle [74] that may enhance the cycle. This is consistent with our observation that *Nautiliales Lebetimonas*-dominated chemolithoautotrophs at WV 65 °C exhibit higher carbon fixation activity (Figs. 2 and 4). The presence of high relative abundances of *narB*, *nasA*, and *nirA* genes in all samples suggests that both *Nautiliales* and *Campylobacterales* may utilize the assimilatory nitrate reduction pathway to obtain NH_4_^+^ (Fig. 4). Although *napA/B* genes, which are involved in dissimilatory nitrate reduction, were present in both *Nautiliales* and *Campylobacterales*, they were only abundant in WV 65 °C (Fig. 4). This observation may be attributed to the fact that higher temperatures, such as in WV 65 °C, are often accompanied by lower oxygen content [1], and nitrate can serve as an alternative electron acceptor in place of oxygen [11]. The absence of genes encoding for dissimilatory nitrite reductase (e.g., *nirBD*, *nrfAH*, *nirS*, *nirK*) but the presence of abundant *napA/B* genes at WV 65 °C (Fig. 4) suggests assimilatory nitrite reduction may be involved in detoxifying nitrite produced by dissimilatory nitrate reductase within cells [75]. *Campylobacterales* could also obtain nitrogen via nitrogen fixation at WV 45 °C and 30 °C (Fig. 4). Overall, the chemoautotrophic members of *Epsilonproteobacteria* employed flexible strategies to acquire inorganic nitrogen for growth in hydrothermal ecosystems characterized by varying physicochemical conditions, including temperature, pH, oxygen levels, and inorganic nitrogen concentrations [76, 77].

Temperature is a crucial factor that influences the oxygen content of water [1]. Indeed, there was a significant decrease in the oxygen content from the reference sites to the interiors of the vents (Fig. 1). Furthermore, particles in vent fluids may contain niches with lower oxygen content because microbes attached to their surfaces can create micro-zones of depleted oxygen through respiration [78, 79]. In this study, we detected three oxidases: cytochrome bd ubiquinol oxidase (Cyd), cytochrome c oxidase cbb3-type (Cco), and aa3-type (Cox) (Fig. 4). Cyd and Cco are expressed in microaerobic conditions, whereas Cox is expressed under aerobic conditions [80, 81]. The Cyd and Cco assigned to *Nautiliales* and *Campylobacterales* were more abundant in the UH fraction, while Cox was much more abundant in the L fraction (Fig. 4), suggesting the *Nautiliales* and *Campylobacterales* experienced microaerobic/anaerobic conditions. One key difference between *Nautiliales* and *Campylobacterales* in terms of oxygen respiration is that *Nautiliales* contain only Cyd, while *Campylobacterales* contain both Cyd and Cco (Fig. 4). We also detected that Cyd is the only oxidase present in all genomes of hyperthermophilic *Aquificae* (Fig. 6). In addition to low oxygen stress, Cco is also capable of oxygen respiration under aerobic conditions [82, 83], while Cyd is involved in the bacterial response to a wide variety of stress conditions, including high temperature and gasotransmitters like H_2_S [84–86]. The highest enrichment and abundance of *cydA/B* genes assigned to *Nautiliales* in the UH fraction of WV 65 °C sample compared to other key genes (Fig. 4) suggest Cyd might play a critical role in enabling *Nautiliales* to thrive in microaerobic/anaerobic conditions induced by high temperature. A previous study also found *cydB* was helpful for *Brucella suis* to grow by utilizing nitrate and detoxifying nitrite [87], which coincides with our findings that *Nautiliales* at WV 65 °C had higher potential to produce nitrite by reducing nitrate compared to the *Campylobacterales* at WV 45 °C and 30 °C.

We were intrigued by the observation that the most abundant KO group enriched at WV 30 °C (Fig. 5a) corresponded to the *TC.FEV.OM* protein, which is an iron (Fe) complex outer-membrane receptor protein. Under oxic conditions, iron (Fe) is primarily present in an oxidized ferric form (Fe^3+^) that is insoluble at neutral pH [88]. To import Fe^3+^, bacteria secrete ferric chelators known as siderophores, which have an intimate relationship with iron complex outer-membrane receptor protein. Thus, the product encoded by the *TC.FEV.OM* gene appeared to play an important role in importing insoluble Fe^3+^ complexes at normal temperatures (e.g. 30 °C). At acidic pH or under anaerobic conditions, iron is predominantly present in a soluble ferrous form (Fe^2+^), which can be directly taken up into the cell via Fe^2+^ transporters like FeoB [89]. A previous study found that Fe^2+^ concentration was much higher in the shallow-sea hydrothermal vent center, and decreased dramatically as the distance from the vent center increased [90]. Since the pH at WV was acidic (Fig. 1) and high temperature usually accompanies low oxygen conditions [1], it is likely that Fe^2+^ served as the primary source of iron for microorganisms at WV 65 °C. Consistent with this hypothesis, the *feoB* gene was found to be one of the most abundant genes, with the highest abundance at WV 65 °C and lowest at WV 30 °C (Fig. 5a). The *TC.FEV.OM* and *feoB* were both relatively abundant at WV 45 °C, indicating both Fe^2+^ and Fe^3+^ were the main iron sources, as Fe^2+^ oxidized to Fe^3+^ when oxygen increased with the decrease in temperature. Our temperature gradient incubation demonstrated that temperature could determine the forms of iron (Fe^2+^ or Fe^3+^) that are available to microbes by affecting oxygen content.

### Microbial adaption strategies of high temperature and low pH

The discussion above reveals the differences in chemolithoautotrophic metabolism of key elements under different temperature and pH conditions. In this session, we further focus on the potential essential functions for chemolithoautotrophs adapting to high temperature and low pH. The comparison of metagenomes from the UH fraction revealed that member transport, signal transduction, and some genes of unknown functions may play important roles for chemolithoautotrophs to adapt to extreme environments (Figs. S7 and S8). For high-temperature adaptation, the gene *rgy*, which is involved in positive supercoiling in closed circular DNA for DNA stability at high temperature [13, 91, 92], was found to be one of the most abundant genes at WV 65 °C, but not detected at WV 30 °C (Fig. 5a). It exists in almost all genomes of *Nautiliales* and *Aquificae* (Fig. 6). Therefore, it is likely to be a key gene for *Nautiliales* living under high temperature. The other genes that facilitated the boom of *Nautiliales* at high temperature may include *proA*, which is involved in the biosynthesis of proline, an amino acid used by thermophiles to keep protein thermostabilization [19], *pepF*, which participates in the regulation of sporulation [93–95], *splB*, which is involved in the repair of UV light-induced DNA damage in spores [96], and *mrcA*, *murG,* and *mltC*, which are involved in biosynthesis of peptidoglycan, the major component of gram-negative cell walls (Fig. 5a). *PepF* only existed in all *Nautiliales* and *splB* only existed in the MAG (WV45 °C bin6) of our study (Fig. 6), indicating sporulation is a special strategy for *Nautiliales* to cope with heat stress, and maintaining UV resistance of spore is a unique strategy for *Nautiliales* inhabiting shallow-sea hydrothermal ecosystem.

Heat shock is a widespread protective mechanism in bacteria that enables them to adapt and survive under adverse conditions. Transcriptional regulation of heat-shock genes can be positive or negative, and mediated by dedicated regulatory proteins. In our study, the genes encoding for dedicated regulatory proteins include *hrcA*, *hspR*, and *rpoH* (Fig. 4), of which *hrcA* and *hspR* are negative regulators, while *rpoH* is a positive regulator [97]. The products of *hrcA* and *hspR* are DNA-binding repressors that can bind specific operators and repress transcription of heat-shock genes under normal conditions and rapidly derepress transcription of these genes upon heat stress [97], while the *rpoH* gene product was able to confer specificity to RNA polymerase in recognizing heat-shock promoters and promote transcription initiation at heat-shock promoters upon heat stress [98]. In our study, *hrcA* was most abundant in WV 65 °C sample, while *hspR* was abundant in all samples (Fig. 4). The activity of HrcA in *Helicobacter pylori* has been proven to be temperature-dependent and become essentially inactive when temperature increased above 37 °C [97]. In this study, we observed that *hrcA* assigned to *Nautiliales* has higher relative abundance in L fraction than in UH/H fractions compared to other key genes assigned to *Nautiliales* at WV 65 °C, which were more abundant in the UH/H fraction (Fig. 4). These results indicate HrcA might be an important thermosensor [99]. The HrcA in *Nautiliales* probably is directly regulated by the master regulator HspR, just like those in *Helicobacter pylori* [97].

For low pH adaption, several genes may play a role in maintaining the cells’ pH homeostasis, including those encoding for the monovalent cation/H^+^ antiporter, Ca_2_^+^/H^+^ antiporter, trk system K^+^ uptake protein, Cu^2+^ exporting ATPase, ABC-2 type transport system permease protein ABC-2.P, which is related to osmotic pressure [100], O-antigen ligase, which catalyzes a key step in the synthesis of lipopolysaccharide (LPS), a matter contributes to the effective permeability barrier of the bacterial outer membrane [101], porin *TC.OOP*, a member of OmpA-OmpF porin that has been suggested to play an important role in acid tolerance [102]. These genes were found to be more abundant at YV 45 °C and 30 °C than at WV 30 °C (Fig. 5c). Notably, the present study identified genes encoding all subunits of proton-pumping NADH: ubiquinone oxidoreductase, also called complex I. The membrane arm subunits of complex I (*nuoA/H/J/K/L/M/N*) were marked as core functions at WV (Fig. S9a). However, these subunits were more abundant at YV 30 °C and 45 °C than at WV 30 °C (Fig. S9b). Especially the *nuoL/M/N* subunits, which are homologous to the Na^+^ or K^+^/H^+^ antiporter family and likely participate in proton translocation [103], were the three most abundant subunits of complex I at YV 30 °C or 45 °C. These results suggest that the membrane arm subunits of complex I may also participate in maintaining cellular pH homeostasis under low pH conditions. In conclusion, the strategies used by *Campylobacterales* at YV to maintain a near-neutral intracellular pH include actively exporting protons with proton pumps, reducing proton influx through electrostatic repulsion by maintaining a positive membrane potential, and forming an impermeable cell membrane to restrict proton influx into the cytoplasm [104]. In addition, genes encoding for the twin-arginine translocation proteins (*tatBC*), which can translocate tightly folded proteins across biological membranes using only a pH gradient independently of ATP [105, 106], were found to be enriched at YV 45 °C and 30 °C (Fig. 5c). This suggests that *Campylobacterales* may take advantage of the extremely acidic condition to conserve energy for metabolism.

## Conclusions

DNA-SIP, combined with 16S rRNA gene and metagenomic high-throughput sequencing, revealed that *Nautiliales* (mainly *Lebetimonas*) were the dominant active chemolithoautotrophs at WV 65 °C, while *Campylobacterales* (mainly *Sulfurimonas* and *Sulfurovum*) actively assimilated DIC under 30 − 45 °C in WV and YV mouths. *Thiotrichales* (mainly *Thiomicrospira*), which was the most abundant taxa in the two vent mouths, did not show significant carbon fixation activity at any of the temperatures tested. The thermophilic *Nautiliales* and mesophilic/psychrophilic *Campylobacterales*, as the two mainly active chemolithoautotrophs in the Kueishantao vents at different temperatures, exhibited unique or preferential pathways in sulfur oxidation, nitrogen acquisition, oxygen utilization, and nitrogen utilization. Compared to *Campylobacterales*, *Nautiliales* that bloomed at WV 65 °C were found to lack the Sox sulfur oxidation system and instead use NAD(H)- rather than NADP(H)-linked glutamate dehydrogenase to catalyze the assimilation of ammonium. They cannot utilize oxygen via the cytochrome c oxidase cbb3-type but have a much higher genetic potential for the activity of cytochrome bd ubiquinol oxidase in oxygen respiration. Additionally, they exhibit a higher genetic potential for increased hydrogen oxidation activity at high temperatures. For high-temperature adaption, *Nautiliales* rely on the gene *rgy* to maintain DNA stability at high temperature, while the gene *splB* is important for maintaining UV resistance of spores in shallow-sea hydrothermal ecosystems by lysing photoproducts. The main strategies utilized by *Campylobacterales* to survive under low pH conditions include (1) exporting protons using proton pumps, (2) reducing proton influx by maintaining a positive membrane potential via electrostatic repulsion, and (3) forming an impermeable cell membrane to restrict proton influx into the cytoplasm. Additionally, notably, the membrane arm subunits of complex I may play a role in regulating cellular pH homeostasis at low pH. In summary, our investigation demonstrates the significant impact of high temperature and low pH on the chemolithoautotrophic microbial compositions and their metabolism of energy and main elements in the hydrothermal vent ecosystem. Moreover, we have identified functional genes that contribute to the adaptation of these microorganisms to such extreme conditions. These findings shed light on the mechanisms and strategies employed by chemolithoautotrophs to survive and thrive in high-temperature and extremely acidic environments.

### Supplementary Information

**Additional file 1: Supplementary figures. Fig. S1. **Geographic location of Kueishantao Islet and geochemical characteristics of the white vent (WV) and yellow vent (YV). **Fig. S2. **Normalized distribution (data scaled between 0 and 1 along the gradient) of bacterial 16S rRNA gene copies in cesium chloride (CsCl) density gradients of temperature gradient incubated samples at the white vent (WV) and yellow vent (YV). The blue, pink, and red bars respectively represent the density range for the light (L, unlabeled), heavy (H, labeled with ^13^C), and ultra-heavy (UH, labeled with both ^13^C and ^15^N) DNA. The triangle, circle, and square symbols represent the fractions that were selected for high-throughput sequencing to obtain bacterial populations that incorporated both NaH^13^CO_3_ and ^15^NH_4_Cl, incorporated only NaH^13^CO_3_, and did not incorporate any labeled substrates, respectively. **Fig. S3. **Quantitative PCR tested bacterial (solid bar) and archaea (hollow bar) abundance in incubated samples and in in situ samples. WV, White vent; YV, yellow vent.** Fig. S4. **Nonmetric multidimensional scaling ordination based on Bray-Curtis dissimilarities among (a) bacterial 16S rRNA gene communities or (b) KEGG functional compositions of the ultra-heavy (UH), heavy (H) and light (L) fractions from temperature gradient incubated samples at White Vent (WV, circle) and Yellow Vent (YV, triangle). Each symbol represents an individual community. **Fig. S5. **Relative abundance of bacterial 16S rRNA gene reads assigned to phylogenetic orders.** Fig. S6. **Comparison of KEGG Orthologys (KOs) among 45 °C at site WV and 30 °C and 45 °C at site YV. In (a), circle size represents the highest normalized relative abundance (RB) of each KO among the three metagenomic libraries. If the difference in relative abundance of a KO was less than two-fold across the three metagenomic libraries, it was noted as a core function (gray circle). If the relative abundance of a KO in one metagenomic library was at least two-fold higher than its abundance in the remaining two libraries, the KO was noted as an enriched (Enr.) function (closed color circles) in that library. Functions with lower relative abundance in one metagenomic library compared to the other two libraries were classified as “diluted (Dil)” KOs (open circles) in that library. In (b), the total relative abundance of each KO category is shown, with the number of KOs in each category indicated above the bars.** Fig. S7. **Composition of core functions from the UH fraction in KEGG level 2 metabolic pathway. (a), comparison among WV 65 °C, WV 45 °C, and WV 30 °C; (b), comparison among YV 45 °C, YV 30 °C, and WV 30 °C. **Fig. S8.** Composition of enriched functions in the KEGG level 2 metabolic pathway from the ultra-heavy (UH) fraction. (a) Comparison among WV 65 °C, WV 45 °C, and WV 30 °C. (b) Comparison among YV 45 °C, YV 30 °C, and WV 30 °C. **Fig. S9.** Ternary plot comparing the abundance of genes encoding all subunits of proton-pumping NADH: ubiquinone oxidoreductase in the ultra-heavy (UH) fractions (a) among 65 °C, 45 °C and 30 °C at site WV, and (b) among WV 30 °C, YV 30 °C, and YV 45 °C. Circle size represents the highest normalized relative abundance (RB) of each gene among the three metagenomic libraries. If the difference in relative abundance of a gene was less than two-fold across the three metagenomic libraries, it was noted as a core gene (gray circle). If the relative abundance of a gene in one metagenomic library was at least two-fold higher than its abundance in the remaining two libraries, the gene was noted as an enriched (Enr.) gene (closed color circles) in that library. Genes with lower relative abundance in one metagenomic library caompared to the other two libraries were classified as “diluted (Dil)” gene (open circles) in that library. **Table S1. **Summary of chemolithoautotrophic MAGs obtained from the white vent metagenomes. **Table S2.** Isolation source, growth temperature and pH condition, and optimum conditions of reference genomes.

## Data Availability

Raw sequencing data of metagenome and the bacterial 16S rRNA gene are available at National Center for Biotechnology Information (NCBI) Sequence Read Archive under BioProject accession number PRJNA979917 with BioSample accession number SAMN35696351 − SAMN35696364 and SAMN35786668 − SAMN35786720. The sequences of MAGs used in genomic phylogenetic tree are available in BioProject accession number PRJNA979917 with BioSample accession number SAMN35790518 − SAMN35790529.
