# Supplementary material for: Strategies of chemolithoautotrophs adapting to high temperature and extremely acidic conditions in a shallow hydrothermal ecosystem
Source: Microbiome. 2023 Dec 5;11:270. doi: 10.1186/s40168-023-01712-w (PMC10696704; doi:10.1186/s40168-023-01712-w)
Supplement: Supplementary file 2 — Additional file 1: Supplementary figures. Fig. S1. Geographic location of Kueishantao Islet and geochemical characteristics of the white vent (WV) and yellow vent (YV). Fig. S2. Normalized distribution (data scaled between 0 and 1 along the gradient) of bacterial 16S rRNA gene copies in cesium chloride (CsCl) density gradients of temperature gradient incubated samples at the white vent (WV) and yellow vent (YV). The blue, pink, and red bars respectively represent the density range for the light (L, unlabeled), heavy (H, labeled with 13C), and ultra-heavy (UH, labeled with both 13C and 15N) DNA. The triangle, circle, and square symbols represent the fractions that were selected for high-throughput sequencing to obtain bacterial populations that incorporated both NaH13CO3 and 15NH4Cl, incorporated only NaH13CO3, and did not incorporate any labeled substrates, respectively. Fig. S3. Quantitative PCR tested bacterial (solid bar) and archaea (hollow bar) abundance in incubated samples and in in situ samples. WV, White vent; YV, yellow vent. Fig. S4. Nonmetric multidimensional scaling ordination based on Bray-Curtis dissimilarities among (a) bacterial 16S rRNA gene communities or (b) KEGG functional compositions of the ultra-heavy (UH), heavy (H) and light (L) fractions from temperature gradient incubated samples at White Vent (WV, circle) and Yellow Vent (YV, triangle). Each symbol represents an individual community. Fig. S5. Relative abundance of bacterial 16S rRNA gene reads assigned to phylogenetic orders. Fig. S6. Comparison of KEGG Orthologys (KOs) among 45 °C at site WV and 30 °C and 45 °C at site YV. In (a), circle size represents the highest normalized relative abundance (RB) of each KO among the three metagenomic libraries. If the difference in relative abundance of a KO was less than two-fold across the three metagenomic libraries, it was noted as a core function (gray circle). If the relative abundance of a KO in one metagenomic library was at least two-fold h [file 40168_2023_1712_MOESM1_ESM.docx]

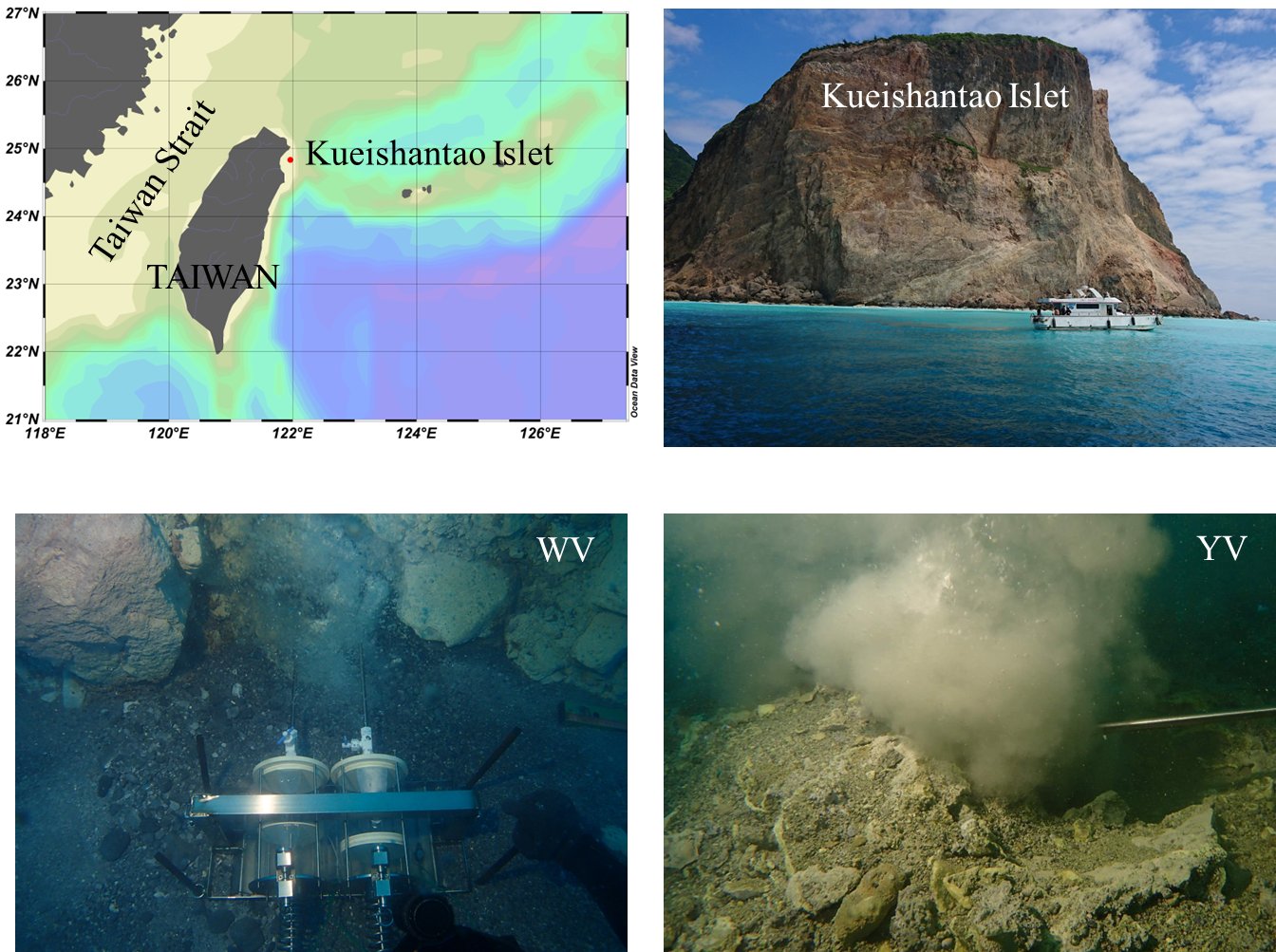


**Fig. S1.** Geographic location of Kueishantao Islet and geochemical characteristics of the white vent (WV) and yellow vent (YV).


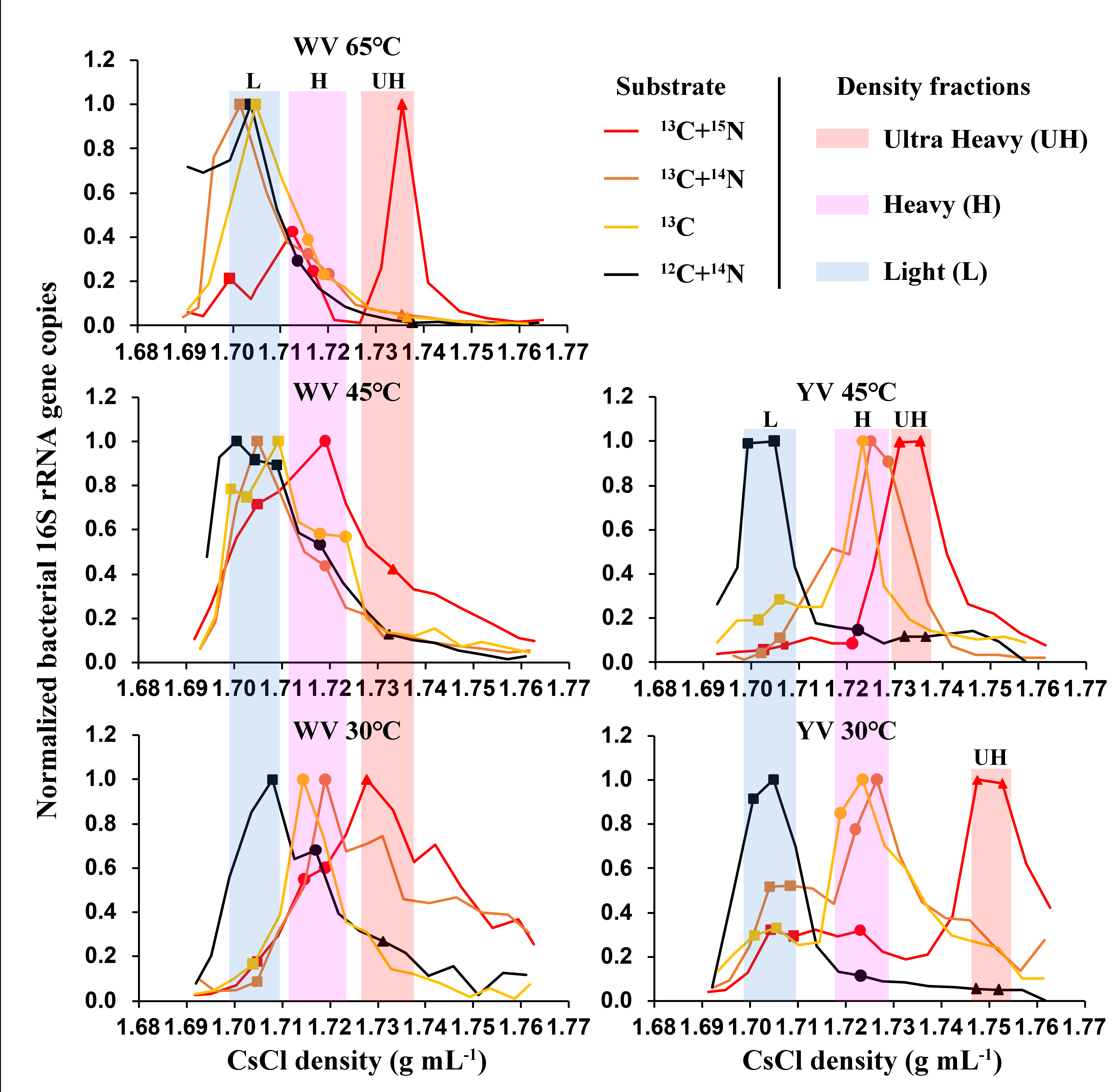


**Fig. S2.** Normalized distribution (data scaled between 0 and 1 along the gradient) of bacterial 16S rRNA gene copies in cesium chloride (CsCl) density gradients of temperature gradient incubated samples at the white vent (WV) and yellow vent (YV). The blue, pink, and red bars respectively represent the density range for the light (L, unlabeled), heavy (H, labeled with ^13^C), and ultra-heavy (UH, labeled with both ^13^C and ^15^N) DNA. The triangle, circle, and square symbols represent the fractions that were selected for high-throughput sequencing to obtain bacterial populations that incorporated both NaH^13^CO_3_ and ^15^NH_4_Cl, incorporated only NaH^13^CO_3_, and did not incorporate any labeled substrates, respectively.


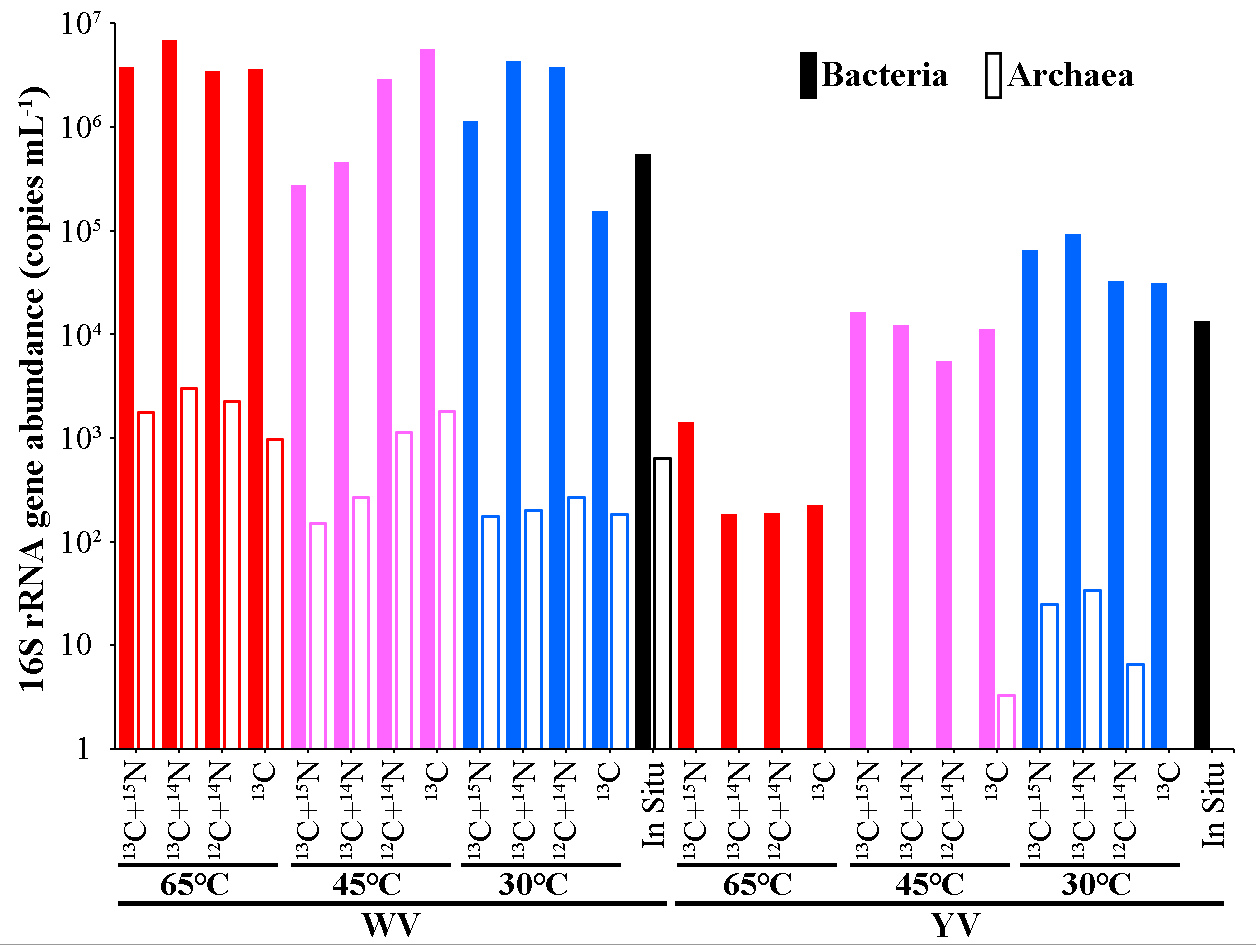


**Fig. S3.** Quantitative PCR tested bacterial (solid bar) and archaea (hollow bar) abundance in incubated samples and in *in situ* samples. WV, White vent; YV, yellow vent.


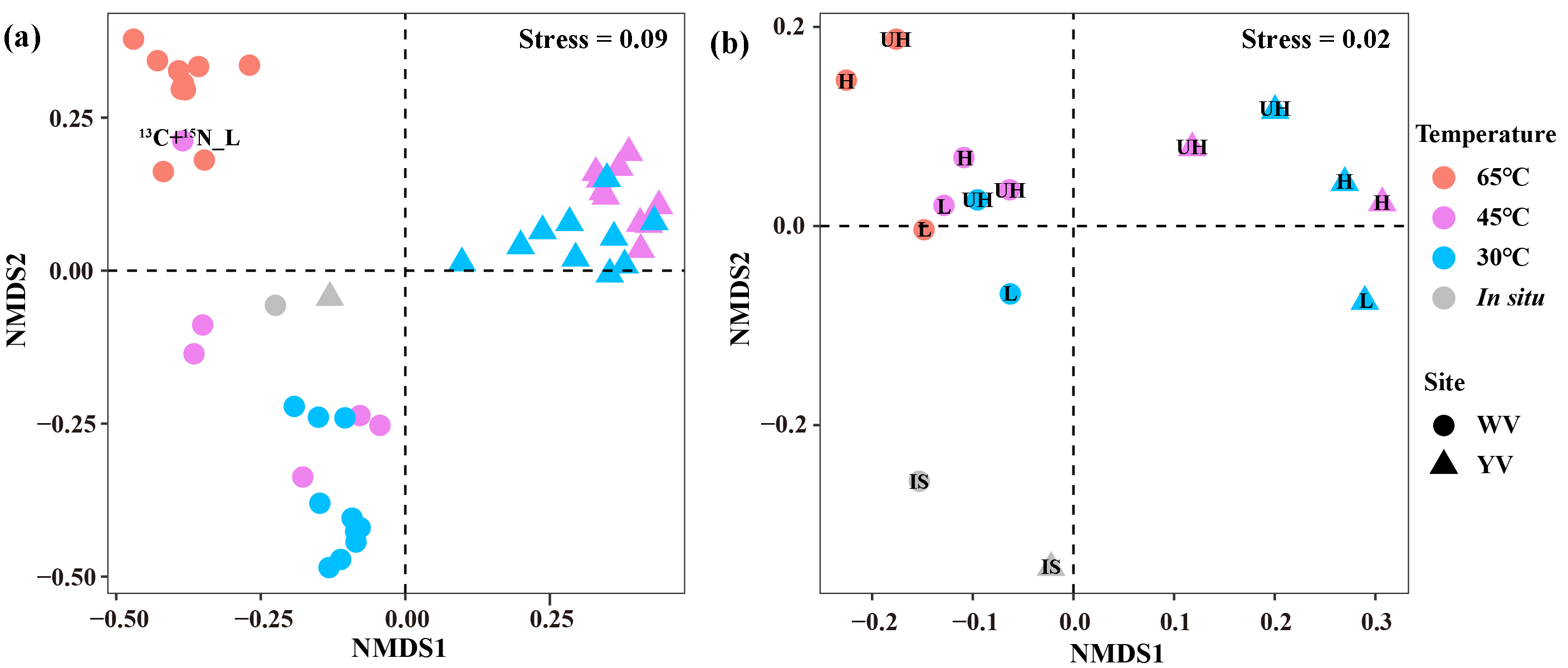


**Fig. S4.** Nonmetric multidimensional scaling ordination based on Bray-Curtis dissimilarities among (a) bacterial 16S rRNA gene communities or (b) KEGG functional compositions of the ultra-heavy (UH), heavy (H) and light (L) fractions from temperature gradient incubated samples at White Vent (WV, circle) and Yellow Vent (YV, triangle). Each symbol represents an individual community.


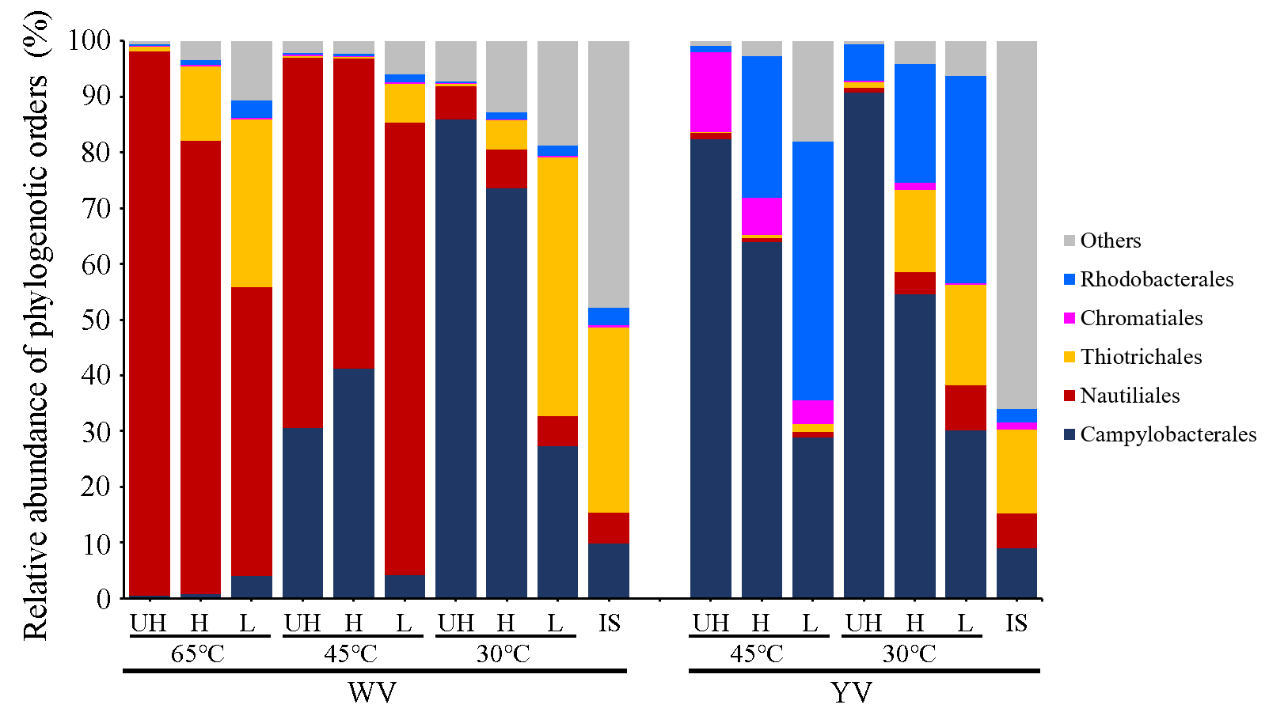


**Fig. S5.** Relative abundance of bacterial 16S rRNA gene reads assigned to phylogenetic orders.


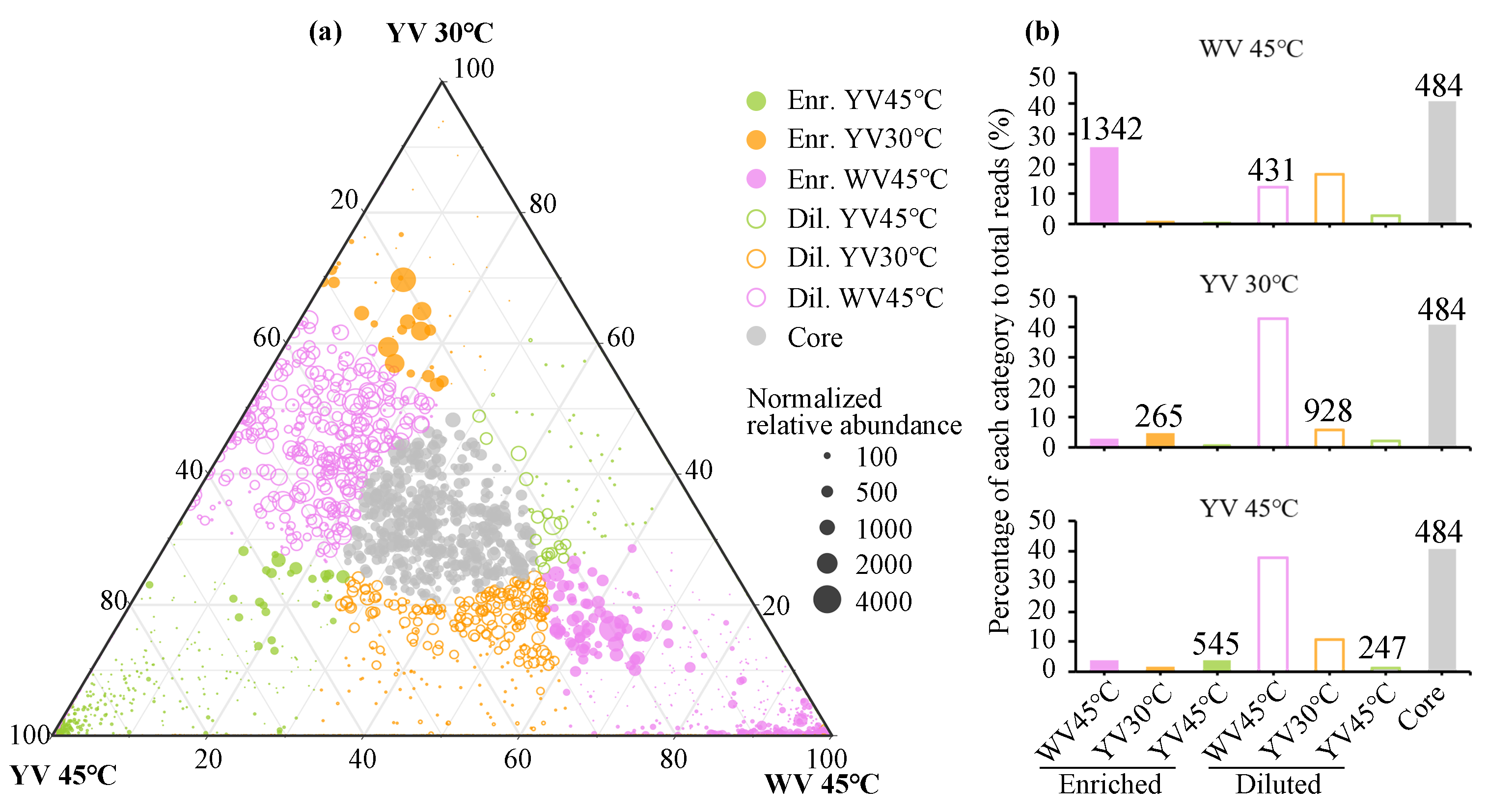


**Fig. S6.** Comparison of KEGG Orthologys (KOs) among 45℃ at site WV and 30℃ and 45℃ at site YV. In (a), circle size represents the highest normalized relative abundance (RB) of each KO among the three metagenomic libraries. If the difference in relative abundance of a KO was less than two-fold across the three metagenomic libraries, it was noted as a core function (gray circle). If the relative abundance of a KO in one metagenomic library was at least two-fold higher than its abundance in the remaining two libraries, the KO was noted as an enriched (Enr.) function (closed color circles) in that library. Functions with lower relative abundance in one metagenomic library compared to the other two libraries were classified as “diluted (Dil)” KOs (open circles) in that library. In (b), the total relative abundance of each KO category is shown, with the number of KOs in each category indicated above the bars.


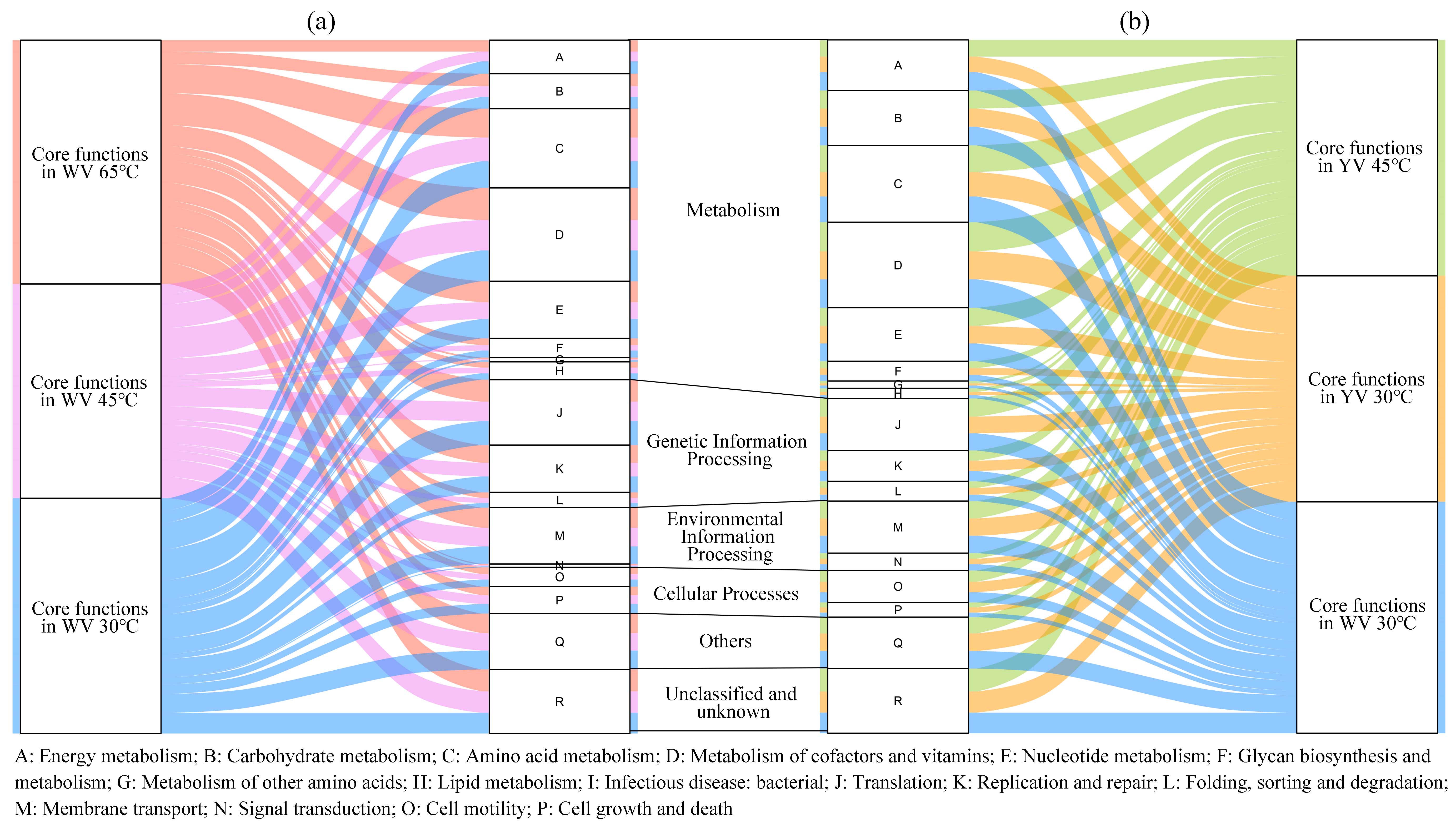


**Fig. S7.** Composition of core functions from the UH fraction in KEGG level2 metabolic pathway. (a), comparison among WV 65℃, WV 45℃, and WV 30℃; (b), comparison among YV 45℃, YV 30℃, and WV 30℃.


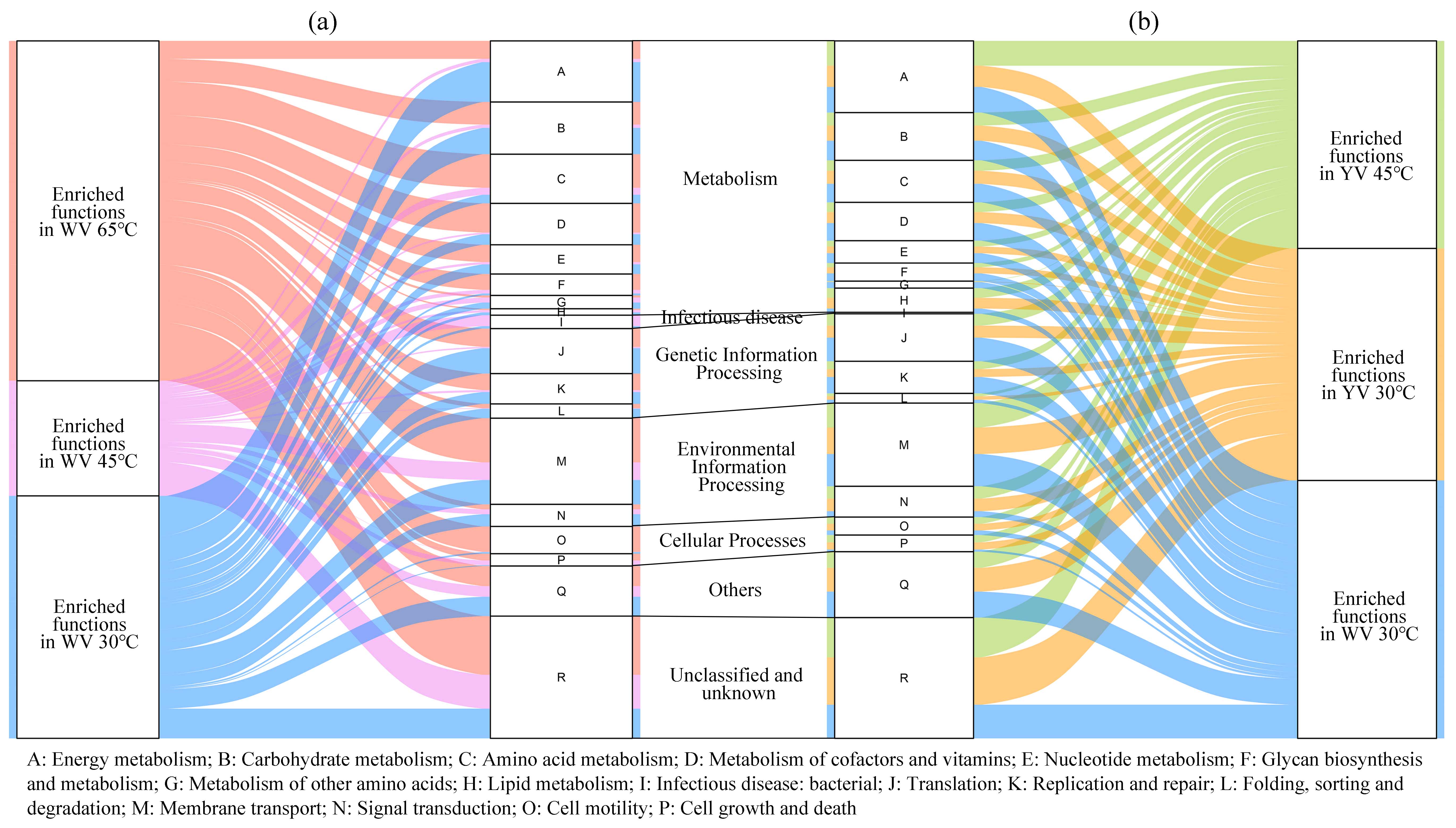


**Fig. S8.** Composition of enriched functions in the KEGG level 2 metabolic pathway from the ultra-heavy (UH) fraction. (a) Comparison among WV 65℃, WV 45℃, and WV 30℃. (b) Comparison among YV 45℃, YV 30℃, and WV 30℃.


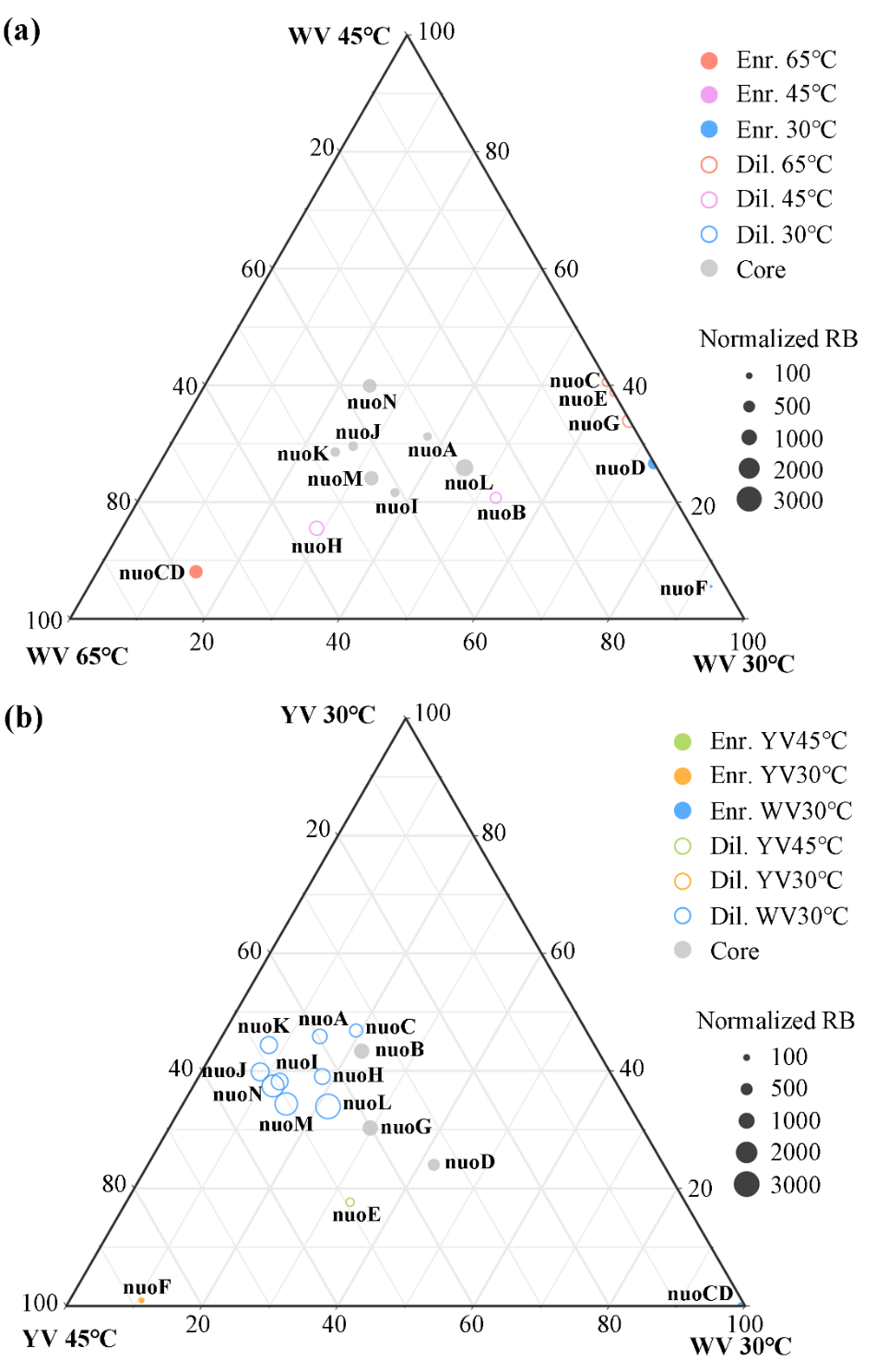


**Fig. S9.** Ternary plot comparing the abundance of genes encoding all subunits of proton-pumping NADH: ubiquinone oxidoreductase in the ultra-heavy (UH) fractions (a) among 65℃, 45℃ and 30℃ at site WV, and (b) among WV 30℃, YV 30℃, and YV 45℃. Circle size represents the highest normalized relative abundance (RB) of each gene among the three metagenomic libraries. If the difference in relative abundance of a gene was less than two-fold across the three metagenomic libraries, it was noted as a core gene (gray circle). If the relative abundance of a gene in one metagenomic library was at least two-fold higher than its abundance in the remaining two libraries, the gene was noted as an enriched (Enr.) gene (closed color circles) in that library. Genes with lower relative abundance in one metagenomic library caompared to the other two libraries were classified as “diluted (Dil)” gene (open circles) in that library.

**Table S1.** Summary of chemolithoautotrophic MAGs obtained from the white vent metagenomes

| Temperature | MAGs | Completeness | Contamination | Taxonomy | Relative abundance（‱） | | |
| --- | --- | --- | --- | --- | --- | --- | --- |
|  |  | (%) | (%) |  | UH | H | L |
| 65℃ | bin4 | 97 | 0.3 | G_Thiomicrospira | 1 | 231 | 1547 |
|  | bin9 | 91.2 | 5 | G_Thermovibrio | 0 | 7 | 40 |
| 45℃ | bin4 | 74.1 | 2.8 | O_Campylobacterales | 4 | 97 | 4 |
|  | bin5 | 72.7 | 1.8 | G_Hydrogenimonas | 0 | 33 | 7 |
|  | bin6 | 99.2 | 2 | O_Nautiliales | 509 | 770 | 6079 |
|  | bin3 | 97.3 | 0.8 | G_Thiomicrospira | 1 | 1 | 429 |
|  | bin8 | 90.3 | 2.7 | G_Thermovibrio | 0 | 0 | 17 |
| 30℃ | bin7 | 91 | 1.2 | G_Sulfurovum | 35 | ND | 2 |
|  | bin9 | 74.6 | 3.3 | G_Hydrogenimonas | 22 | ND | 0 |
|  | bin15 | 51.5 | 3.8 | G_Nitratifractor | 348 | ND | 9 |
|  | bin5 | 96.7 | 0 | G_Thiomicrospira | 1 | ND | 904 |
|  | bin8 | 98.1 | 2.9 | G_Thermovibrio | 0 | ND | 25 |

UH, ultra-heavy density fraction; H, heavy density fraction; L, light density fraction; O, order; G, genus; ND, no data.

**Table S2.** Isolation source, growth temperature and pH condition, and optimum conditions of reference genomes

| Strain | Source of isolation | Growth temperature (℃) | Optimum temperature (℃) | Growth pH | Optimum pH | References |
| --- | --- | --- | --- | --- | --- | --- |
| Cetia pacifica DSM27783 | deep-sea HV | 45-75 | 55-60 | 4.5-7.5 | 5.5-6.0 | Grosche et al. (2015) |
| Caminibacter mediatlanticus TB-2 | deep-sea HV | 45-75 | 55 | 4.5-7.5 | 5.5 | Voordeckers et al. (2005) |
| Nautilia sp.PV 1 | deep-sea HV | ND | 55 | ND | ND | NCBI |
| Nautilia profundicola AmH | deep-sea HV | 35-50 | 40 | 6.0-9.0 | 7 | Smith et al. (2008) |
| Lebetimonas natsushimae HS1857 | deep-sea HV | 35-65 | 55 | 5.5-7.1 | 6.4 | Nagata et al. (2017) |
| Lebetimonas acidiphila JH292 | deep-sea HV | ND | ND | ND | ND | Meyer et al. (2014) |
| Lebetimonas acidiphila JS085 | deep-sea HV | ND | ND | ND | ND | Meyer et al. (2014) |
| Lebetimonas acidiphila JH369 | deep-sea HV | ND | ND | ND | ND | Meyer et al. (2014) |
| Nitratiruptor tergarcus DSM 16512 | deep-sea HV | 40-57 | 55 | 5.4-6.9 | 6.4 | Nakagawa et al. (2005) |
| Hydrogenimonas thermophila EP1-55-1 | deep-sea HV | 35-65 | 55 | 4.9-7.2 | 5.9 | Takai et al. (2004) |
| Nitratifractor salsuginis DSM 16511 | deep-sea HV | 28-40 | 37 | 5.6-7.6 | 7 | Nakagawa et al. (2005) |
| Sulfurovum ithotrophicum ATCC BAA-797 | deep-sea HV | 10-40 | 28-30 | 5.0-9.0 | 6.5-7.0 | Inagaki et al. (2004) |
| Sulfurovum riftiae 1812E | deep-sea HV | 25-45 | 35 | 5.0-8.0 | 6 | Giovannelli et al. (2016) |
| Sulfuricurvum kujiense DSM 16994 | crude-oil storage cavity | 10-35 | 25 | 5.0-8.0 | 7 | Kodama and Watanabe (2004) |
| Sulfurimonas autotrophica DSM 16294 | deep-sea HV | 10-40 | 23-26 | 4.5-9.0 | 6.0-7.5 | Inagaki et al. (2003) |
| Sulfurimonas denitrificans DSM 1251 | deep-sea HV | 4-35 | 30 | 5.4-8.6 | 6.1 | Takai et al. (2006) |
| Sulfurimonas gotlandica GD1 | redox zone  of sea | 4-20 | 15 | 6.5-8.4 | 6.7-8.0 | Labrenz et al. (2013) |
| Sulfurimonas hongkongensis AST-10 | coastal sediment | 15-35 | 30 | 6.5-8.5 | 7.0-7.5 | Cai et al. (2014) |
| Desulfurobacterium thermolithotrophum DSM 11699 | deep-sea HV | 40-75 | 70 | 4.4-7.5 | 6 | L’Haridon et al. (1998) |
| Thermovibrio guaymasensis DSM 15521 | deep-sea HV | 50-88 | 75-80 | 5.5-7.5 | 6.0-6.2 | L’Haridon et al. (2006) |
| Thermovibrio ammonificans HB-1 | deep-sea HV | 60-80 | 75 | 5-7 | 5.5 | Vetriani et al. (2004) |
| Balnearium lithotrophicum DSM 16304 | deep-sea HV | 45-80 | 70-75 | 5.0-7.0 | 5.4 | Takai et al. (2003) |
| Thiomicrospira pelophila DSM 1534 | coastal mud flat | 3.5-42 | 28-30 | 5.6-9 | 7 | Kuenen and Veldkamp (1972) |
| Thiomicrospira microaerophila ASL8-2 | soap lake | ND | 25-28 | 8-10 | 9 | Boden et al. (2017) |
| Thiomicrospira aerophila AL3 | soda lake | ND-40 | ND | 7.5-10.6 | 9.8-10 | Boden et al. (2017) |
| Thiomicrospira cyclica ALM1 | Mono Lake | ND | ND | 7.5-10.5 | 9.5 | Boden et al. (2017) |
| Hydrogenovibrio kuenenii DSM 12350 | intertidal mud flat | 3.5-42 | 29-33.5 | 4.0-7.5 | 6 | Brinkhoff et al. (1999) |
| Thiomicrorhabdus indica 13-15A | deep-sea HV | 10-45 | 28 | 5.0-9.0 | 7 | Liu et al. (2020) |
| Thiomicrorhabdus chilensis DSM 12352 | intertidal mud flat | 3.5-42 | 32-37 | 5.3-8.5 | 7 | Liu et al. (2020) |
| Thiomicrorhabdus aquaedulcis HaS4 | Harutori Lake | 0-25 | 22 | 6.2-8.8 | 6.6-7.4 | Kojima and Fukui (2019) |
| Thiomicrorhabdus arctica DSM 13458 | coastal sediments | -2-20.8 | 11.5-13.2 | 6.5-9.0 | 7.3-8.0 | Knittel et al. (2005) |

ND, no data; HV, hydrothermal vent.

**References**

Boden, R., Scott, K. M., Williams, J., Russel, S., Antonen, K., Rae, A. W., & Hutt, L. P. (2017). An evaluation of Thiomicrospira, Hydrogenovibrio and Thioalkalimicrobium: reclassification of four species of Thiomicrospira to each Thiomicrorhabdus gen. nov. and Hydrogenovibrio, and reclassification of all four species of Thioalkalimicrobium to Thiomicrospira. International Journal of Systematic and Evolutionary Microbiology, 67(5), 1140-1151.

Brinkhoff, T., Muyzer, G., Wirsen, C. O., & Kuever, J. (1999). Thiomicrospira kuenenii sp. nov. and Thiomicrospira frisia sp. nov., two mesophilic obligately chemolithoautotrophic sulfur-oxidizing bacteria isolated from an intertidal mud flat. International Journal of Systematic and Evolutionary Microbiology, 49(2), 385-392.

Cai, L., Shao, M. F., & Zhang, T. (2014). Non-contiguous finished genome sequence and description of Sulfurimonas hongkongensis sp. nov., a strictly anaerobic denitrifying, hydrogen-and sulfur-oxidizing chemolithoautotroph isolated from marine sediment. Standards in Genomic Sciences, 9, 1302-1310.

Giovannelli, D., Chung, M., Staley, J., Starovoytov, V., Le Bris, N., & Vetriani, C. (2016). Sulfurovum riftiae sp. nov., a mesophilic, thiosulfate-oxidizing, nitrate-reducing chemolithoautotrophic epsilonproteobacterium isolated from the tube of the deep-sea hydrothermal vent polychaete Riftia pachyptila. International journal of systematic and evolutionary microbiology, 66(7), 2697-2701.

Grosche, A., Sekaran, H., Pérez-Rodríguez, I., Starovoytov, V., & Vetriani, C. (2015). Cetia pacifica gen. nov., sp. nov., a chemolithoautotrophic, thermophilic, nitrate-ammonifying bacterium from a deep-sea hydrothermal vent. International journal of systematic and evolutionary microbiology, 65(Pt_4), 1144-1150.

Inagaki, F., Takai, K., Nealson, K. H., & Horikoshi, K. (2004). Sulfurovum lithotrophicum gen. nov., sp. nov., a novel sulfur-oxidizing chemolithoautotroph within the ε-Proteobacteria isolated from Okinawa Trough hydrothermal sediments. International Journal of Systematic and Evolutionary Microbiology, 54(5), 1477-1482.

Knittel, K., Kuever, J., Meyerdierks, A., Meinke, R., Amann, R., & Brinkhoff, T. (2005). Thiomicrospira arctica sp. nov. and Thiomicrospira psychrophila sp. nov., psychrophilic, obligately chemolithoautotrophic, sulfur-oxidizing bacteria isolated from marine Arctic sediments. International Journal of Systematic and Evolutionary Microbiology, 55(2), 781-786.

Kojima, H., & Fukui, M. (2019). Thiomicrorhabdus aquaedulcis sp. nov., a sulfur-oxidizing bacterium isolated from lake water. International journal of systematic and evolutionary microbiology, 69(9), 2849-2853.

Kuenen, J. G., & Veldkamp, H. (1972). Thiomicrospira pelophila, gen. n., sp. n., a new obligately chemolithotrophic colourless sulfur bacterium. Antonie van Leeuwenhoek, 38, 241-256.

Labrenz, M., Grote, J., Mammitzsch, K., Boschker, H. T., Laue, M., Jost, G., ... & Jürgens, K. (2013). Sulfurimonas gotlandica sp. nov., a chemoautotrophic and psychrotolerant epsilonproteobacterium isolated from a pelagic redoxcline, and an emended description of the genus Sulfurimonas. International journal of systematic and evolutionary microbiology, 63(Pt 11), 4141.

L’Haridon, S., Cilia, V., Messner, P., Raguenes, G., Gambacorta, A., Sleytr, U. B., ... & Jeanthon, C. (1998). Desulfurobacterium thermolithotrophum gen. nov., sp. nov., a novel autotrophic, sulphur-reducing bacterium isolated from a deep-sea hydrothermal vent. International Journal of Systematic and Evolutionary Microbiology, 48(3), 701-711.

L'Haridon, S., Reysenbach, A. L., Tindall, B. J., Schönheit, P., Banta, A., Johnsen, U., ... & Jeanthon, C. (2006). Desulfurobacterium atlanticum sp. nov., Desulfurobacterium pacificum sp. nov. and Thermovibrio guaymasensis sp. nov., three thermophilic members of the Desulfurobacteriaceae fam. nov., a deep branching lineage within the Bacteria. International journal of systematic and evolutionary microbiology, 56(12), 2843-2852.

Liu, X., Jiang, L., Hu, Q., Lyu, J., & Shao, Z. (2020). Thiomicrorhabdus indica sp. nov., an obligately chemolithoautotrophic, sulfur-oxidizing bacterium isolated from a deep-sea hydrothermal vent environment. International Journal of Systematic and Evolutionary Microbiology, 70(1), 234-239.

Meyer, J. L., & Huber, J. A. (2014). Strain-level genomic variation in natural populations of Lebetimonas from an erupting deep-sea volcano. The ISME Journal, 8(4), 867-880.

Nagata, R., Takaki, Y., Tame, A., Nunoura, T., Muto, H., Mino, S., ... & Nakagawa, S. (2017). Lebetimonas natsushimae sp. nov., a novel strictly anaerobic, moderately thermophilic chemoautotroph isolated from a deep-sea hydrothermal vent polychaete nest in the Mid-Okinawa Trough. Systematic and Applied Microbiology, 40(6), 352-356.

Nakagawa, S., Takai, K., Inagaki, F., Horikoshi, K., & Sako, Y. (2005). Nitratiruptor tergarcus gen. nov., sp. nov. and Nitratifractor salsuginis gen. nov., sp. nov., nitrate-reducing chemolithoautotrophs of the ε-Proteobacteria isolated from a deep-sea hydrothermal system in the Mid-Okinawa Trough. International journal of systematic and evolutionary microbiology, 55(2), 925-933.

Smith, J. L., Campbell, B. J., Hanson, T. E., Zhang, C. L., & Cary, S. C. (2008). Nautilia profundicola sp. nov., a thermophilic, sulfur-reducing epsilonproteobacterium from deep-sea hydrothermal vents. International journal of systematic and evolutionary microbiology, 58(7), 1598-1602.

Takai, K., Nakagawa, S., Sako, Y., & Horikoshi, K. (2003). Balnearium lithotrophicum gen. nov., sp. nov., a novel thermophilic, strictly anaerobic, hydrogen-oxidizing chemolithoautotroph isolated from a black smoker chimney in the Suiyo Seamount hydrothermal system. International Journal of Systematic and Evolutionary Microbiology, 53(6), 1947-1954.

Takai, K., Nealson, K. H., & Horikoshi, K. (2004). Hydrogenimonas thermophila gen. nov., sp. nov., a novel thermophilic, hydrogen-oxidizing chemolithoautotroph within the ε-Proteobacteria, isolated from a black smoker in a Central Indian Ridge hydrothermal field. International Journal of Systematic and Evolutionary Microbiology, 54(1), 25-32.

Takai, K., Suzuki, M., Nakagawa, S., Miyazaki, M., Suzuki, Y., Inagaki, F., & Horikoshi, K. (2006). Sulfurimonas paralvinellae sp. nov., a novel mesophilic, hydrogen-and sulfur-oxidizing chemolithoautotroph within the Epsilonproteobacteria isolated from a deep-sea hydrothermal vent polychaete nest, reclassification of Thiomicrospira denitrificans as Sulfurimonas denitrificans comb. nov. and emended description of the genus Sulfurimonas. International Journal of Systematic and Evolutionary Microbiology, 56(8), 1725-1733.

Vetriani, C., Speck, M. D., Ellor, S. V., Lutz, R. A., & Starovoytov, V. (2004). Thermovibrio ammonificans sp. nov., a thermophilic, chemolithotrophic, nitrate-ammonifying bacterium from deep-sea hydrothermal vents. International Journal of Systematic and Evolutionary Microbiology, 54(1), 175-181.

Voordeckers, J. W., Starovoytov, V., & Vetriani, C. (2005). Caminibacter mediatlanticus sp. nov., a thermophilic, chemolithoautotrophic, nitrate-ammonifying bacterium isolated from a deep-sea hydrothermal vent on the Mid-Atlantic Ridge. International Journal of Systematic and Evolutionary Microbiology, 55(2), 773-779.
